# Supplementary material for: Thalamic Drive of Cortical Parvalbumin-Positive Interneurons during Down States in Anesthetized Mice
Source: Curr Biol. 2019 May 6;29(9):1481–1490.e6. doi: 10.1016/j.cub.2019.04.007 (PMC6509281; doi:10.1016/j.cub.2019.04.007)
Supplement: Document S1. Figures S1–S4 [file mmc1.pdf]

Current Biology, Volume *29*

Supplemental Information

Thalamic Drive of Cortical Parvalbumin-Positive Interneurons during Down States in Anesthetized Mice

Stefano Zucca, Valentina Pasquale, Pedro Lagomarsino de Leon Roig, Stefano Panzeri, and Tommaso Fellin

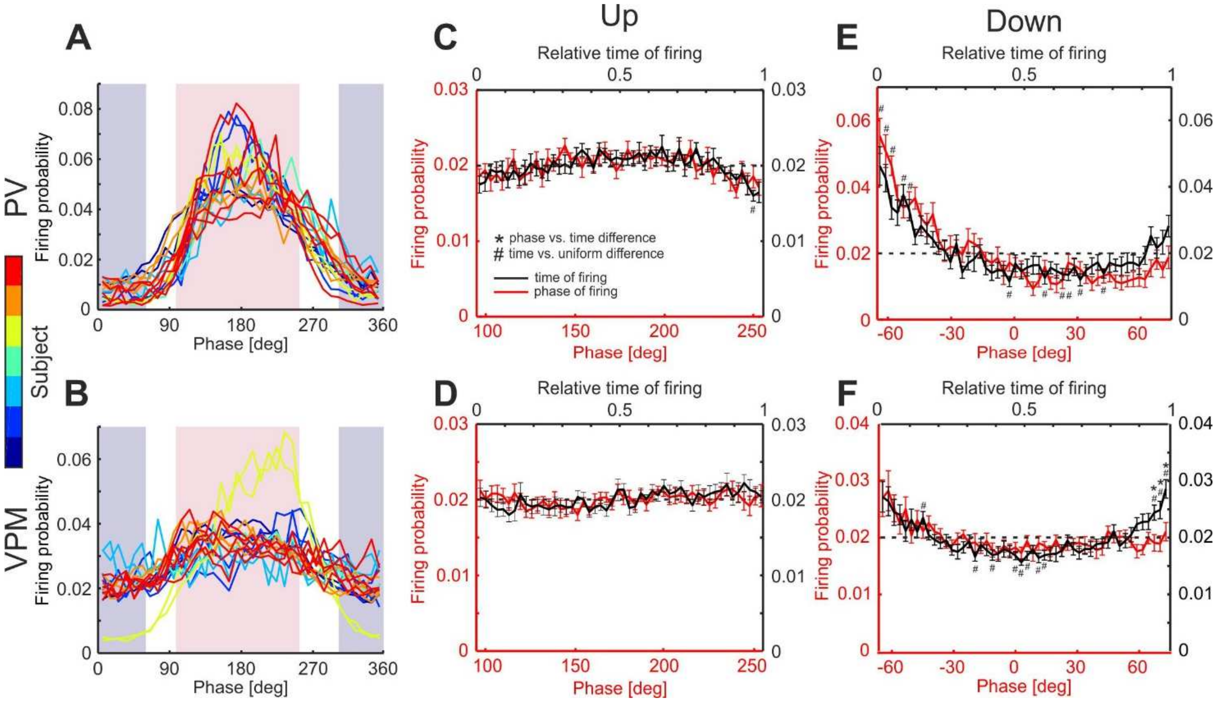

Figure S1. Phase-of-firing and relative time-of-firing distributions of PV cells and VPM MUA during cortical up- and down-states. Related to Figure 1. (A-B) Phase-of-firing distributions of all PV interneurons (color-coded by animal (A) and simultaneously recorded VPM MUA (B). **(C-D)** Phase-of-firing distribution (red, mean ± s.e.m., n = 18 cells in 7 animals) and relative time-of-firing distribution (black) for PV interneurons (C) and VPM MUA (D) during cortical up-states. **(E-F**) Same as for (C, D), but for cortical down-states (mean ± s.e.m., n = 18 recordings in 7 animals). For all panels: asterisks indicate point-wise significant statistical differences between the two curves (paired Student's t-test, FDR corrected for multiple comparisons, p-level = 0.05). Hashtags indicate point-wise significant statistical differences between time-of-firing distributions and the uniform distribution (one-sample Student's t-test, FDR corrected for multiple comparisons, p-level = 0.05).

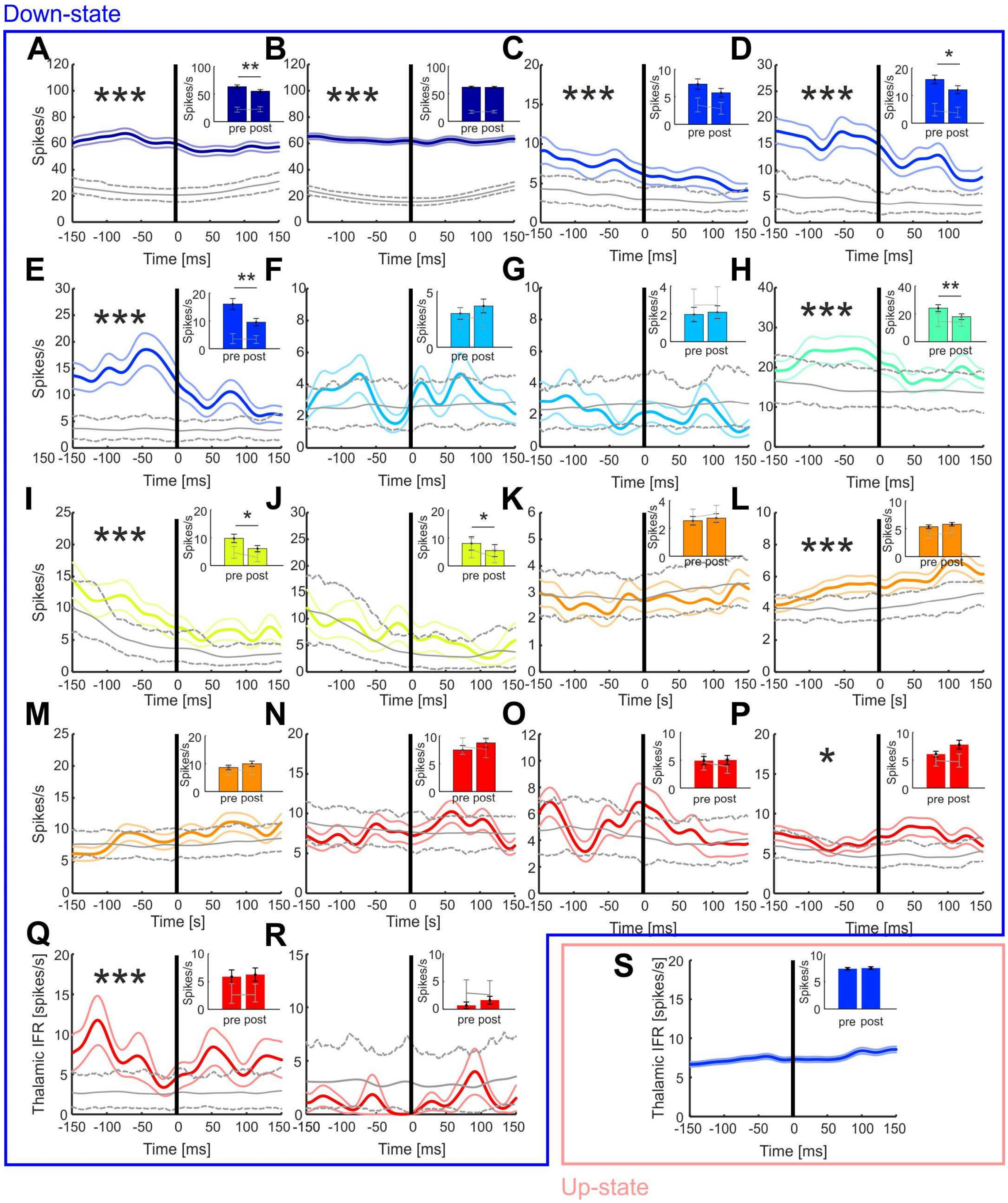

Figure S2. Spike-triggered average analysis across individual experiments. Related to Figure 2. (A-R) Spike-triggered VPM firing rate based on PV interneuron spike times during cortical down-states for all recorded cells (n = 18 from 7 animals, Gaussian kernel standard deviation 12.5 ms, mean ± s.e.m.). Statistical difference of the pre-spike VPM mean firing rate with respect to surrogate data is indicated by the asterisks (z-test, $p < 0.05$). Insets: VPM firing rate before and after a PV spike in down-state (pre: [-100, 0] ms; post: [0, 100 ms]; mean ± s.e.m.; Wilcoxon signed rank test). **(S)** Same as in (A-R**)** but for PV interneuron spike times during cortical up-states for one representative recorded cell.

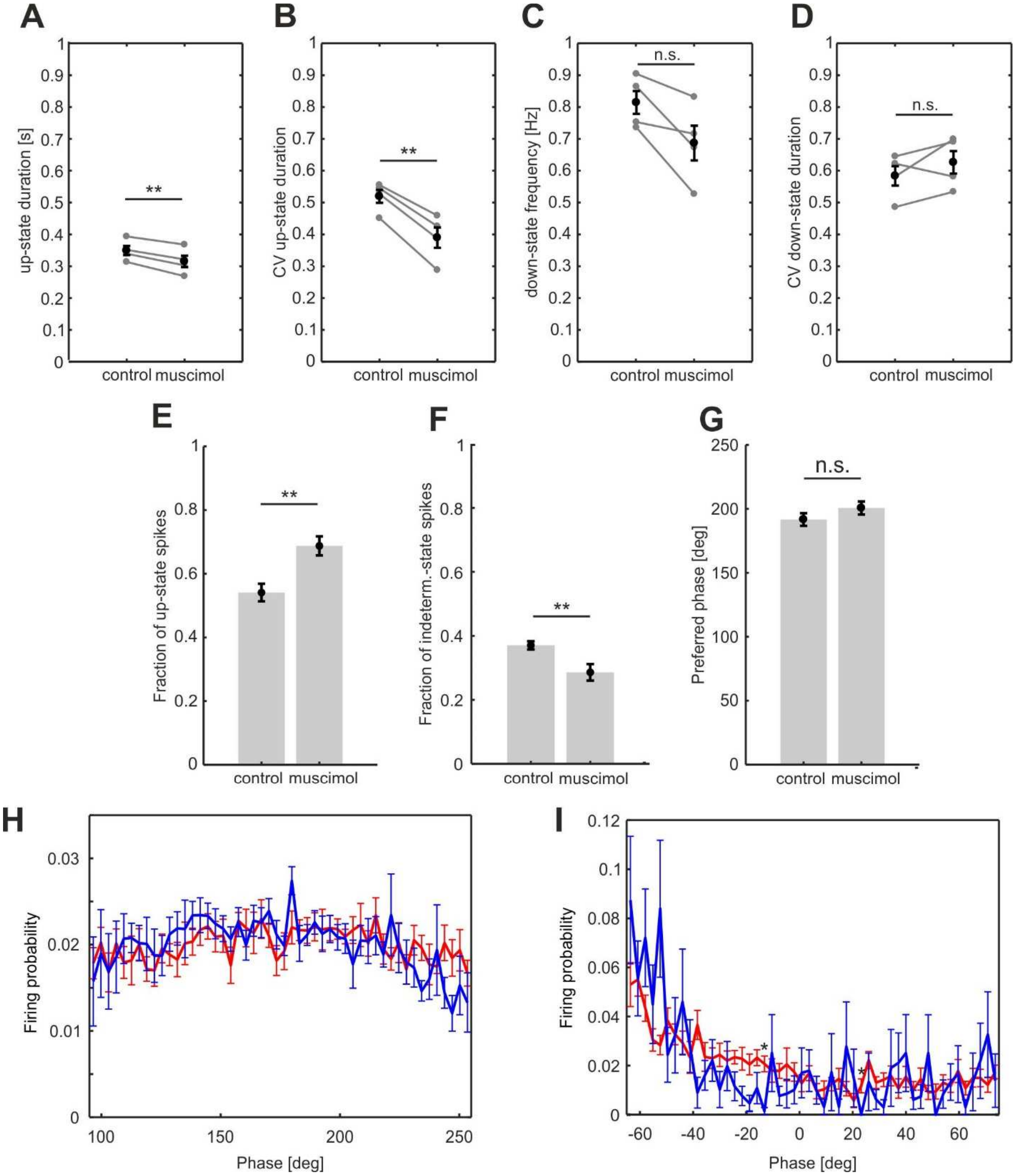

Figure S3. Effect of thalamic muscimol injection on cortical up and down-state dynamics and PV interneuron firing activity. **Related to Figure 3. (A-B**) Up-state duration (A) and its coefficient of variation (B) under control conditions and after muscimol injection in the thalamus (mean ± s.e.m., paired Student's t-test, p = 0.009 for A and p = 0.003 for B, n = 4 animals). **(C**) Down-state frequency under control conditions and after muscimol injection in the thalamus (mean ± s.e.m., p = 0.056, paired Student's t-test, n = 4 animals). **(D**) Coefficient of variation of down-state duration under control conditions and after muscimol injection in the thalamus (mean ± s.e.m., paired Student's t-test p = 0.27 for D, n = 4 animals). **(E-F**) Fraction of up (E) and indeterminate (F) state spikes under control conditions and after muscimol injection (mean ± s.e.m., Student's t-test, p = 0.003 for C and p = 0.008 for D, control: n = 8 cells, muscimol: n = 7 cells from 4 animals). **(G**) Mean phase of firing of PV interneurons under control conditions and after thalamic muscimol injection (circular mean ± s.e.m, p = 0.26, Watson-Williams test, control: n = 8, muscimol: n = 7 from 4 animals). **(H-I**) Phase-of-firing distribution of PV interneurons during cortical up (H) and down (I) states under control conditions (red, mean ± s.e.m., n = 8 cells in 4 animals) and after thalamic muscimol injection (blue, mean ± s.e.m., n = 7 cells in 4 animals). In both panels, the two curves do not show significant point-wise differences, except for two points (marked by asterisks, Student's t-test, FDR corrected for multiple comparisons, p-level = 0.05).

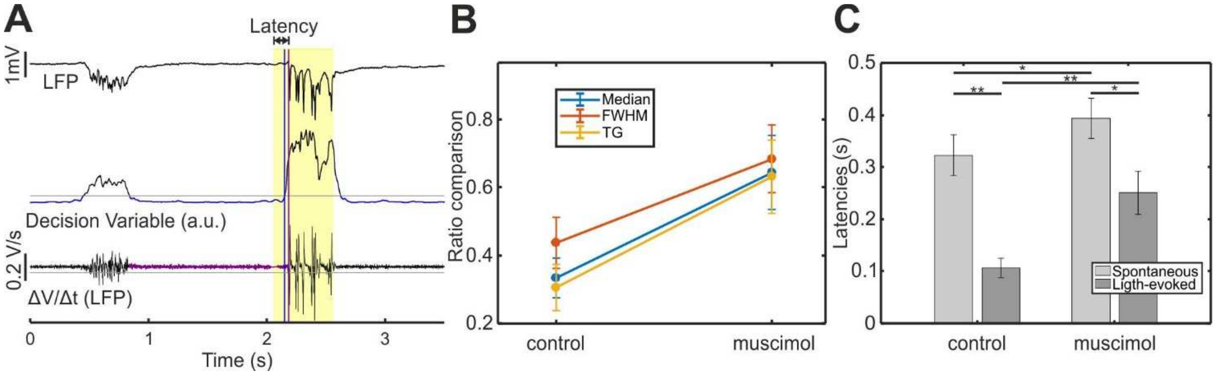

Figure S4. Latencies of down-to-up transitions upon inhibition of cortical PV interneurons under control condition and after thalamic pharmacological inactivation. Related to Figure 4. (**A**) Representative cortical LFP trace after muscimol injection in the thalamus (top) showing characteristic silent and active periods during slow oscillation network dynamics. The gamma-filtered signal (middle) is used to detect down-states (blue periods). This detection is then used to select the traces in which the beginning of the optogenetic inhibition of PV cells (yellow bar) occurred in a down-state. The transition to an up-state (purple vertical line) is defined as occurring when the LFP temporal derivative (bottom) crosses the threshold T (gray line). The latency of the transition is therefore the time interval between the beginning of the yellow bar and the purple vertical line. (**B**) Comparison of the average latency ratios calculated using three different thresholds (median, full-width-half-maximum and two-Gaussian mixture fitting, indicated by different colors) for down-state detection (see STAR Methods) (mean ± s.e.m., one-way ANOVA, p = 0.36 for control and p = 0.94 for muscimol, n = 7 animals). **(C)** Latencies of spontaneous and light-evoked down-to-up transitions under control conditions and after muscimol application in the thalamus (mean ± s.e.m., n = 7 animals).
