# Supplementary material for: Thalamic Drive of Cortical Parvalbumin-Positive Interneurons during Down States in Anesthetized Mice
Source: Curr Biol. 2019 May 6;29(9):1481–1490.e6. doi: 10.1016/j.cub.2019.04.007 (PMC6509281; doi:10.1016/j.cub.2019.04.007)
Supplement: Document S2. Article plus Supplemental Information [file mmc2.pdf]

Article

Current Biology

Thalamic Drive of Cortical Parvalbumin-Positive Interneurons during Down States in Anesthetized Mice

Graphical Abstract

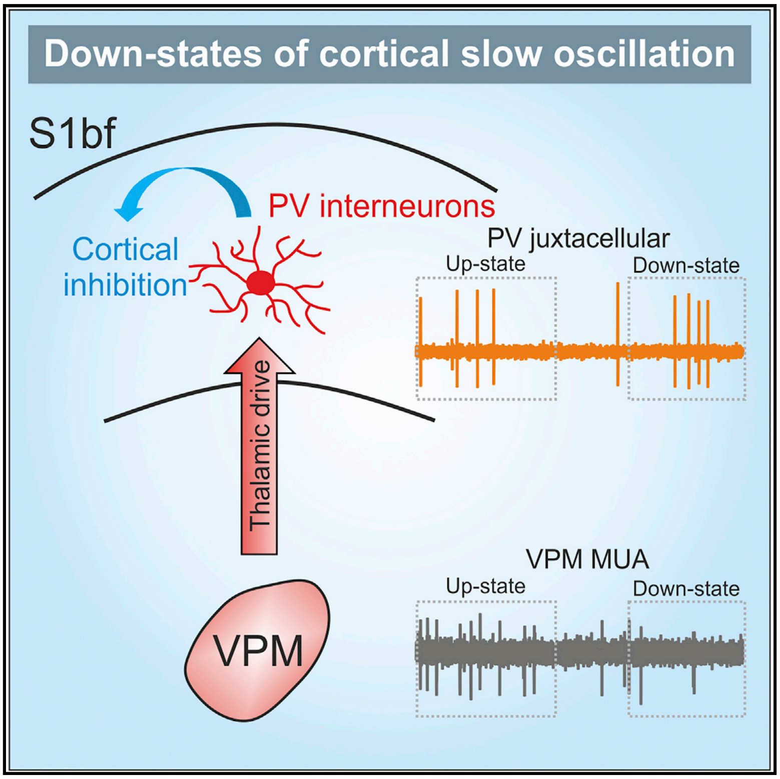

Highlights

- Thalamic activity in VPM is associated with longer down states in S1bf
- Thalamic inactivation decreases spikes of PV cells in S1bf during down states
- Thalamic suppression delays network response to PV cell silencing in down states
- Thalamus suppresses S1bf during down states through activation of PV cells

Authors

Stefano Zucca, Valentina Pasquale, Pedro Lagomarsino de Leon Roig, Stefano Panzeri, Tommaso Fellin

Correspondence

tommaso.fellin@iit.it

In Brief

Using a combination of electrophysiology, local pharmacology, and cell-specific optogenetic perturbations, Zucca et al. provide evidence that spontaneous thalamic activity inhibits cortex during down states through the suprathreshold activation of PV-positive interneurons.

Zucca et al., 2019, Current Biology 29, 1481–1490
May 6, 2019 © 2019 The Authors. Published by Elsevier Ltd.
https://doi.org/10.1016/j.cub.2019.04.007

Current Biology
Article

CellPress

Thalamic Drive of Cortical Parvalbumin-Positive Interneurons during Down States in Anesthetized Mice

Stefano Zucca,[1,2,4] Valentina Pasquale,[1,2,4] Pedro Lagomarsino de Leon Roig,[1,2] Stefano Panzeri,[2,3] and Tommaso Fellin[1,2,5,*]
[1]Optical Approaches to Brain Function Laboratory, Department of Neuroscience and Brain Technologies, Istituto Italiano di Tecnologia, Via Morego 30, 16163 Genova, Italy
[2]Neural Coding Laboratory, Istituto Italiano di Tecnologia, Via Morego 30, 16163 Genova, Italy
[3]Neural Computation Laboratory, Center for Neuroscience and Cognitive Systems at UniTn, Istituto Italiano di Tecnologia, Corso Bettini 31, 38068 Rovereto, Italy
[4]These authors contributed equally
[5]Lead Contact
*Correspondence: tommaso.fellin@iit.it
https://doi.org/10.1016/j.cub.2019.04.007

SUMMARY

Up and down states are among the most prominent features of the thalamo-cortical system during non-rapid eye movement (NREM) sleep and many forms of anesthesia. Cortical interneurons, including parvalbumin (PV) cells, display firing activity during cortical down states, and this GABAergic signaling is associated with prolonged down-state durations. However, what drives PV interneurons to fire during down states remains unclear. We here tested the hypothesis that background thalamic activity may lead to suprathreshold activation of PV cells during down states. To this aim, we performed two-photon guided juxtasomal recordings from PV interneurons in the barrel field of the somatosensory cortex (S1bf) of anesthetized mice, while simultaneously collecting the local field potential (LFP) in S1bf and the multi-unit activity (MUA) in the ventral posteromedial (VPM) thalamic nucleus. We found that activity in the VPM was associated with longer down-state duration in S1bf and that down states displaying PV cell firing were associated with increased VPM activity. Moreover, thalamic inhibition through application of muscimol reduced the fraction of spikes discharged by PV cells during cortical down states. Finally, we inhibited PV interneurons using optogenetics during down states while monitoring cortical LFP under control conditions and after thalamic muscimol injection. We found increased latency of the optogenetically triggered down-to-up transitions upon thalamic pharmacological blockade compared to controls. These findings demonstrate that spontaneous thalamic activity inhibits cortex during down states through the activation of PV interneurons.

INTRODUCTION

Slow-wave oscillations represent the dominant cortical rhythm observed during deep stages of non-rapid eye movement (NREM) sleep and under several types of anesthesia [1–6]. These oscillations are characterized by the rhythmic alternation (0.2–1 Hz) of silent (down) and active (up) network states, which can be captured in the local field potential (LFP) signal as depth-positive and depth-negative waves, respectively [7]. Down and up states occur in the cortex and in many subcortical regions, including the thalamus [1, 8], and they are believed to be crucial in the regulation of several processes, such as memory consolidation, sensory responses, and synaptic plasticity [9–18]. During up states, most types of cortical neurons display depolarized membrane potential and, in some cases, action potential firing. In contrast, down states are characterized by hyperpolarized membrane potential and no action potential firing in most cortical cells, including principal cells [19, 20]. However, some types of GABAergic interneurons, e.g., parvalbumin (PV)-positive fast spiking cells, were found to spike during down states [21, 22], and the speed of the LFP phase was observed to decrease after PV cell spikes during down states [22]. Moreover, optogenetic inhibitory manipulation of PV interneurons during down states reliably triggered swift up-state transitions, indicating that the spiking activity of PV cells during these cortical silent states was crucial to prolong down-state duration [22] and to maintain cortical networks in the silent state. However, what drives PV interneurons to spike during cortical down states remains unknown.

Here, we combined electrophysiological recording together with cell-specific optogenetic manipulation and pharmacology in anesthetized mice to demonstrate that thalamic activity significantly contributes to drive PV cells spiking during cortical down states. We found that activity in the thalamic ventral posteromedial (VPM) nucleus was associated with longer down-state duration in S1bf and that down states during which PV cells fired were associated with increased VPM spiking. Importantly, injection of muscimol in the thalamus decreased spikes of PV cells in the cortex during down states. Moreover, it increased the latency

This is an open access article under the CC BY license (http://creativecommons.org/licenses/by/4.0/).

of up-state transitions triggered by optogenetic inhibition of PV cells during down states. Altogether, our data demonstrate that spontaneous thalamic firing causes cortical silencing through the recruitment of PV interneurons during cortical silent states.

RESULTS

Simultaneous Recording of VPM and Cortical PV Cell Activity during Up and Down States

To simultaneously monitor cortical PV cells and VPM network activity, we performed two-photon-targeted juxtasomal recordings of PV interneurons in S1bf of anesthetized transgenic animals while collecting the LFP in S1bf and the multi-unit activity (MUA) in the VPM thalamic nucleus (Figure 1A). Transgenic animals were obtained by crossing the PV-cre mouse line and the TdTomato reporter line (see STAR Methods). We identified up and down states (Figure 1B), combining information about the LFP phase in the delta (<4 Hz) frequency band [7] and the power in higher bandwidths (i.e., beta and low gamma [10, 50] Hz) [23]. This method reliably detects up and down states from the LFP (false positive rates ~4%) [22]. In Figure 1B and in all other figures, time periods identified as up or down states are shown as pink or purple shaded areas, respectively.

PV-positive interneurons were mainly active during up states but also fired during down states (Figure 1C), in agreement with previous reports [21, 22]. The ratio of PV-active up states (i.e., the number of up states in which the interneuron fired divided by the total number of detected up states) was higher than the ratio of PV-active down states (Figure 1D), and the mean spike count per active up state was higher than the mean spike count per active down state (Figure 1E). In the VPM, we also observed a higher fraction of spikes during up states compared to down states (Figure 1F), although the difference in VPM activity between up and down states was smaller compared to that observed for PV interneurons (difference between fraction of up and down-state spikes: PV 0.459 ± 0.028, VPM 0.160 ± 0.035, paired Student's t test, $p = 1.42E-7$, n = 18 in 7 animals). The ratios of VPM-active up and down states were also different (Figure 1G). The mean VPM spike count per active state was higher in up states compared to down states (Figure 1H). PV spikes were not uniformly distributed during the up and down-state cycle (Rayleigh test for non-uniformity of phase-of-firing distributions; $p < 0.01$) with most spikes distributed in the up state phase range ([95, 255] degrees; Figures 1I and 1J) and less spikes in the down-state phases ([295, 75] degrees; Figures 1I and S1A). VPM MUA was phase locked to the up and down-state cycle in 6 out of 7 animals, corresponding to 16 out of 18 PV-cell recordings (Rayleigh test for non-uniformity of circular data; $p < 0.01$; Figures 1K and 1L), although the locking strength of VPM was lower than that of PV interneurons (paired Student's t test; $p = 1.47E-6$; n = 16 in 6 animals). Figure S1 shows the normalized phase of firing distributions limited to either up (Figures S1C and S1D) or down (Figures S1E and S1F) states for PV (Figures S1A, S1C, and S1E) and VPM (Figures S1B, S1D, and S1F) recordings with the corresponding relative time of firing distributions (mean ± SEM; n = 18 cells in 7 animals; see STAR Methods).

VPM Activity Correlates with Down-State Duration and PV Firing in Down States

Given that VPM neurons were active during down states, we asked whether VPM contributes driving cortical PV interneurons spiking during cortical down states. To this aim, we first correlated VPM MUA with the duration of simultaneously recorded cortical down states. We found that down-state duration was longer when the VPM was active (VPM active; Figure 2A) compared to down-state duration recorded when the VPM was silent (VPM silent; Figure 2A). Moreover, firing of PV interneurons during down states was associated to higher VPM spike count (Figure 2B), supporting the idea that VPM may promote interneuron spiking during cortical down states.

We next characterized the temporal relationship between VPM spikes and PV spikes during down states. If VPM neurons contribute to drive PV cells to fire, then VPM neurons should tend to fire earlier than PV cells, whereas a causal effect of PV firing on VPM MUA signal would lead to the opposite temporal relationship. To address this issue, we computed the instantaneous firing rate (IFR) of VPM MUA triggered on the timing of PV spikes in down states (Figure 2C). To investigate whether PV cell firing probability in down states can be correlated to an increase of VPM firing rate, we compared the spike-triggered VPM IFR with the one computed from randomly sampled time instants taken from PV-silent down states ("surrogate" PV spikes; Figure 2C). Surrogates were constructed preserving the phase of firing distribution in the down state, the maximal number of spikes per down state, and the total number of sampled down states (see STAR Methods for details). We examined the temporal relationship between VPM and PV firing for each individual PV cell. Figures S2A–S2R report the real (mean ± SEM) and surrogate (mean ± [5–95] percentile range) spike-triggered VPM IFR functions for all recorded PV interneurons (n = 18 from 7 animals). In Figure 2D, we reported the *Z* scores of VPM firing rate with respect to surrogate data distribution in 100-ms time windows preceding and following PV spikes in down state for each experiment. In 10 out of 18 PV cells, VPM firing frequency in the pre-spike window was significantly higher than expected by chance (false discovery rate [FDR] correction applied). Although heterogeneity of response was observed in different cells, the finding that 10 significant cells out of 18 at $p < 0.05$ was highly significant at the population level (binomial test; $p = 1.12E-10$; n = 18 recordings from 7 animals). Moreover, at the single-PV cell level, the pre-spike VPM mean firing rate was higher (one-tailed Wilcoxon signed-rank test; FDR corrected) than the post-spike VPM mean firing rate (see STAR Methods) in 6 out of 18 recordings (see Figure 2E for a representative cell), and no experiment showed a post-spike VPM firing rate higher than the pre-spike firing rate. Although heterogeneity was present across cells, the finding that 6 individual cells had significantly higher VPM firing rate in the pre-spike time window was highly significant (binomial test; $p = 1.1E-6$; n = 18 recordings from 7 animals). Finally, we normalized both pre- and post-spike VPM firing rate to the overall mean VPM firing rate triggered by surrogate spikes to account for different baseline VPM firing across experiments. We found higher percentages of variations in the pre-spike window compared to the post-spike time window (Figure 2F). Taken together, all results suggest

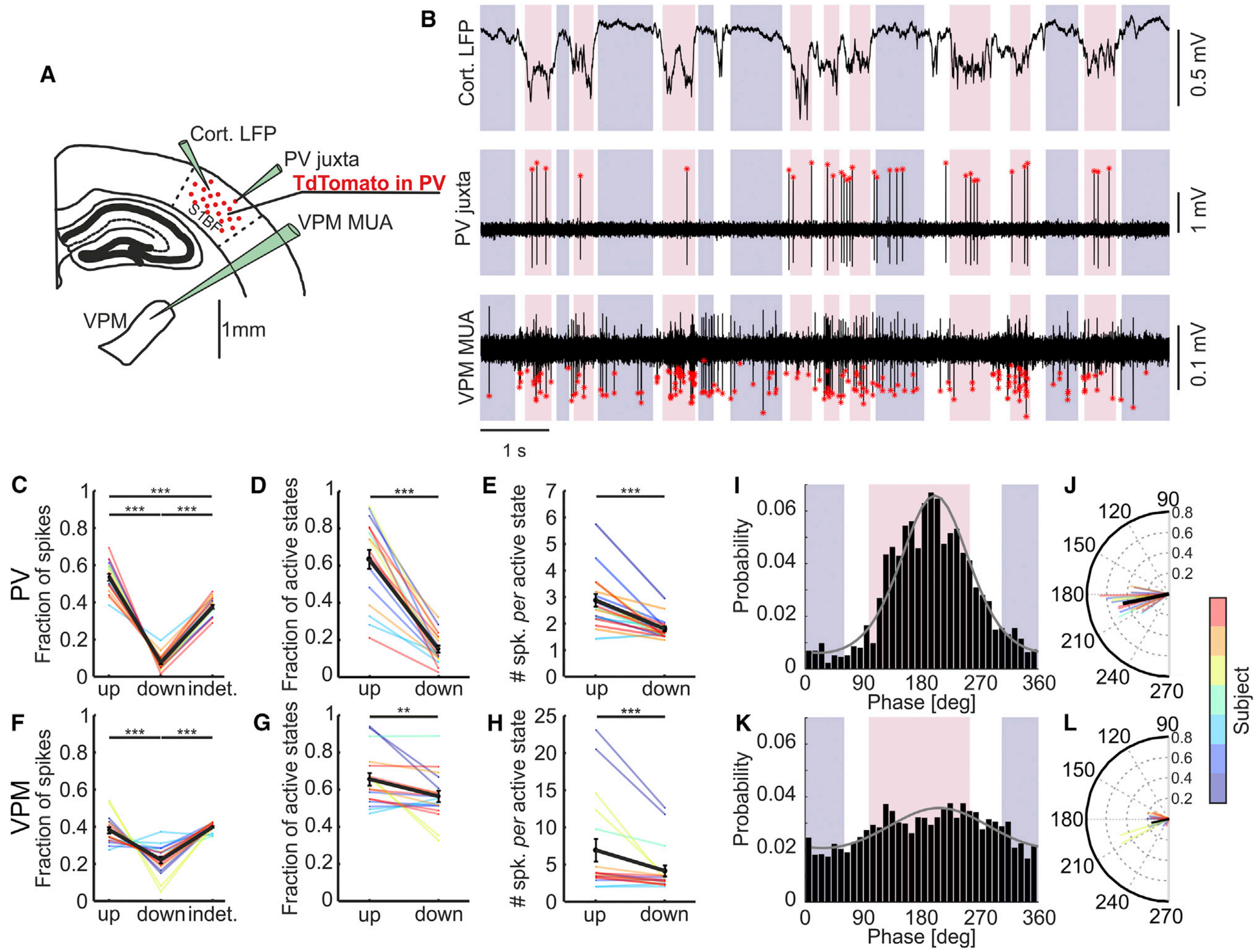

Figure 1. Firing of Cortical PV Interneurons and Thalamic VPM Nucleus during Up and Down States

(A) Schematic of the experimental configuration. Two-photon guided juxtasomal recordings of PV interneurons in S1bf were conducted while recording the LFP in S1bf and the MUA in the VPM.

(B) Representative traces of cortical LFP (top), single-unit activity from a PV interneuron (middle), and thalamic MUA (bottom) during up and down states. Pink and purple in the background indicate up and down states identified from the LFP, respectively. White indicates indeterminate states (see STAR Methods). Red asterisks mark detected spikes.

(C) Fraction of action potentials fired by PV cells in up, down, and indeterminate periods (up-down $p = 2.8E{-}11$; up-indeterminate $p = 0.00015$; down-indeterminate $p < 2E{-}16$; paired Student's t test; Bonferroni corrected; n = 18 cells from 7 animals) relative to the total number of PV spikes. In this as well in other figures, colored dots and lines identify individual cells. Black dots and lines indicate the average value across cells. Error bars indicate SEM. Not significant (n.s.), $p > 0.05$; *$p < 0.05$; **$p < 0.01$; ***$p < 0.001$.

(D) Fraction of active up states and active down states relative to the total number of up and down states, respectively ($p = 4.65E{-}9$; paired Student's t test; n = 18 cells from 7 animals).

(E) Average number of spikes fired by PV cells per single up or down state ($p = 2.82E{-}5$; paired Student's t test; n = 18 cells from 7 animals).

(F–H) Same as in (C)–(E) but for thalamic MUA. In (F), up-down $p = 0.00086$, up-indeterminate $p = 0.95020$, down-indeterminate $p = 4.7E{-}7$; paired Student's t test; Bonferroni corrected; n = 18 recordings from 7 animals. In (G), $p = 0.008$; paired Student's t test; n = 18 recordings from 7 animals. In (H), $p = 7.98E{-}4$; Wilcoxon signed-rank test; n = 18 recordings from 7 animals.

(I) Phase of firing distribution for one representative PV interneuron. Pink and purple indicate the range of phases corresponding to up and down states, respectively. The gray line shows the von Mises fit (see STAR Methods; $\mu = 188^\circ$ and $\kappa = 1.38$). The preferred phase of firing (see STAR Methods) was 192 ± 13 degrees (circular mean ± angular deviation; n = 18 cells in 7 animals) and the locking strength 0.461 ± 0.006 (n = 18 cells in 7 animals).

(J) Circular plot of the mean phase of firing of PV interneurons. The thick black line indicates the overall mean phase of firing and average locking strength (n = 18 cells from 7 animals).

(K and L) Phase of firing distribution for one representative VPM MUA (K) and circular plot of the mean phase of firing for all recorded VPM MUA signals (L). VPM MUA was simultaneously recorded together with the cortical PV interneuron. The preferred phase of firing of the 16 out of 18 phase-locked VPM MUA recordings was 191 ± 23 degrees (n = 16 cells in 6 animals), not different from that of simultaneously recorded PV interneurons (Watson-Williams test; $p = 0.92$; n = 16 in 6 animals).

See also Figure S1.

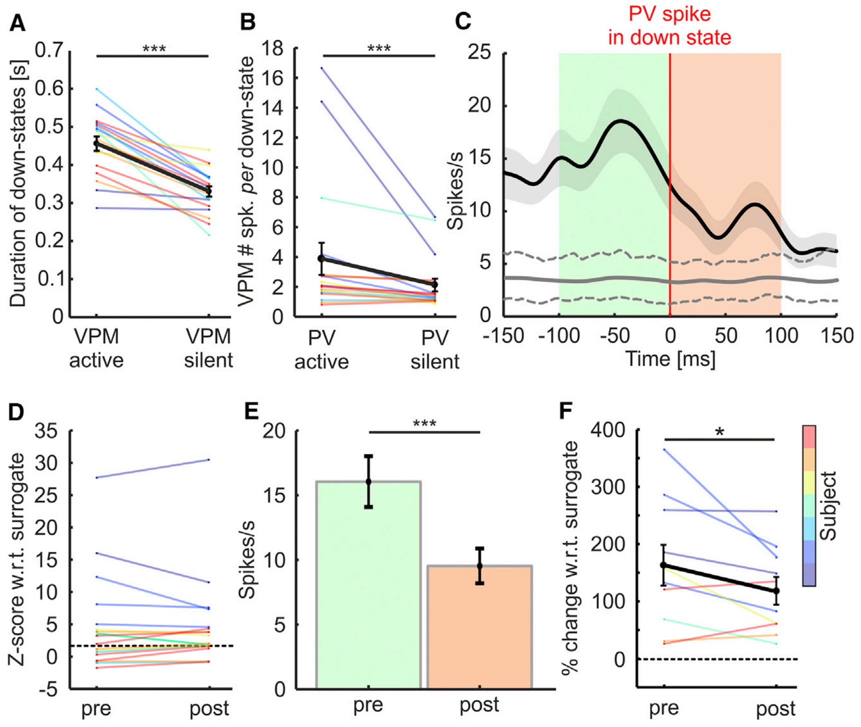

Figure 2. VPM Firing Correlates with Cortical Down-State Duration and PV Firing in Down States

(A) Duration of cortical down states associated with detected VPM activity (VPM-active) and VPM inactivity (VPM-silent; p = 4.5E−7; one-tailed paired Student's t test; n = 18 recordings from 7 animals).

(B) VPM spike count in PV-active and PV-silent cortical down states (p = 2.5E−4; one-tailed Wilcoxon signed rank test; n = 18 cells in 7 animals).

(C) Spike-triggered VPM IFR based on the timing of PV spikes recorded during cortical down states for one representative recording (Gaussian kernel SD, 12.5 ms; the thick black line indicates the mean; the shaded area the SEM). The VPM IFR triggered by "surrogate" spikes is displayed with the thick gray line. Dotted gray lines indicate the 5th and 95th percentiles of the distribution of the mean surrogate spike-triggered VPM IFR (100 surrogates).

(D) *Z* scores in the pre- and post-time windows of the PV-spike-triggered VPM mean firing rate with respect to surrogate data: 10 out of 18 recordings showed a significant difference between actual and surrogate data in the pre-spike window (one-tailed z-test). The dashed line indicates significance at p = 0.05. Cells with *Z* scores corresponding to p > 0.05 lie above the dashed line and are marked with asterisks in Figure S2.

(E) Mean firing rate of the VPM before and after PV spikes in down states (pre: [−100, 0] ms; post: [0, 100 ms]; mean ± SEM; p = 3E−4; two-sided Wilcoxon signed rank test; n = 116 spikes for the same recording as in C).

(F) Variation of VPM mean firing rate relative to the overall mean surrogate firing rate in the pre- and post- windows (p = 0.032; one-tailed Student's t test; n = 10 cells from 6 animals) for those cells showing a firing rate significantly higher than that of the surrogate data.

See also Figure S2.

that the occurrence of VPM spikes in the down state increases the probability of PV spikes in down states shortly after.

To check whether the observed temporal relationship between VPM and PV spikes is specific to the down state, we also computed the VPM IFR triggered on the timing of PV spikes in the up state (Figure S2S). We compared the pre- and post-spike VPM mean firing rate for each PV cell (one-tailed Wilcoxon signed-rank test; FDR corrected) during up states, and we found 1 cell out of 18 in which the pre-spike VPM firing rate was higher than the post-spike (binomial test; p = 0.2265; n = 18 recordings from 7 animals). We also found 2 cells out of 18 in which the pre-spike VPM firing rate was lower than the post-spike one (binomial test; p = 0.0581; n = 18 recordings from 7 animals). Therefore, in contrast to what we observed during down state, no temporal relationship between VPM and PV spikes emerges during up states at the population level.

Thalamic Inactivation Reduces PV Spikes during Down States

The observation of a statistical dependency between the firing of different cells (VPM neurons and PV cells in this case; Figure 2) is not enough to prove a causal relationship between firing of the corresponding two cellular populations [24]. To directly test for the causal necessity of the thalamus in the regulation of PV cell firing during down states, we used a perturbative pharmacological approach to silence the thalamus. We applied the GABA receptor agonist muscimol locally in the thalamus, and we performed two-photon-targeted juxtasomal recordings of PV interneurons in S1bf while collecting cortical LFP in S1bf and the MUA in the VPM thalamic nucleus before and after the pharmacological manipulation (Figure 3A). Muscimol application almost completely abolished spiking activity in the VPM MUA (10.12 ± 3.86 spikes/s under control conditions versus 0.03 ± 0.01 spikes/s after muscimol application; n = 11 animals; Wilcoxon signed-rank test; p = 9.77E−4; Figure 3B), and *a posteriori* observation in fixed tissue confirmed local application of muscimol in the thalamus (see STAR Methods). Muscimol application induced a reduction in the frequency of up states (Figure 3C) and an increase in down-state duration (Figure 3D), consistently with the previous literature [25]. Up-state duration (Figure S3A) and its coefficient of variation (Figure S3B) were also decreased. Down-state frequency (Figure S3C) and the coefficient of variation of down state duration (Figure S3D) were not significantly changed.

Most importantly, we found that the fraction of PV spikes in down states was decreased upon pharmacological inactivation of the thalamus (Figure 3E), in agreement with the hypothesis that the VPM contributes to drive PV cells spiking during down states. At the same time, the fraction of PV spikes in the up state increased (Figure S3E), the fraction of spikes in the indeterminate state decreased (Figure S3F), and the mean firing rate of PV cells remained constant (3.4 ± 1.0 spikes/s under control conditions, n = 8, versus 2.5 ± 0.5 spikes/s after muscimol application, n = 7; Student's t test; p = 0.47). The average preferred phase of firing of PV cells after muscimol injection was 201 ± 5 degrees (Figure S3G; n = 7 cells in 4 animals), not different from that under control conditions (192 ± 5 degrees; n = 8 cells in 4 animals;

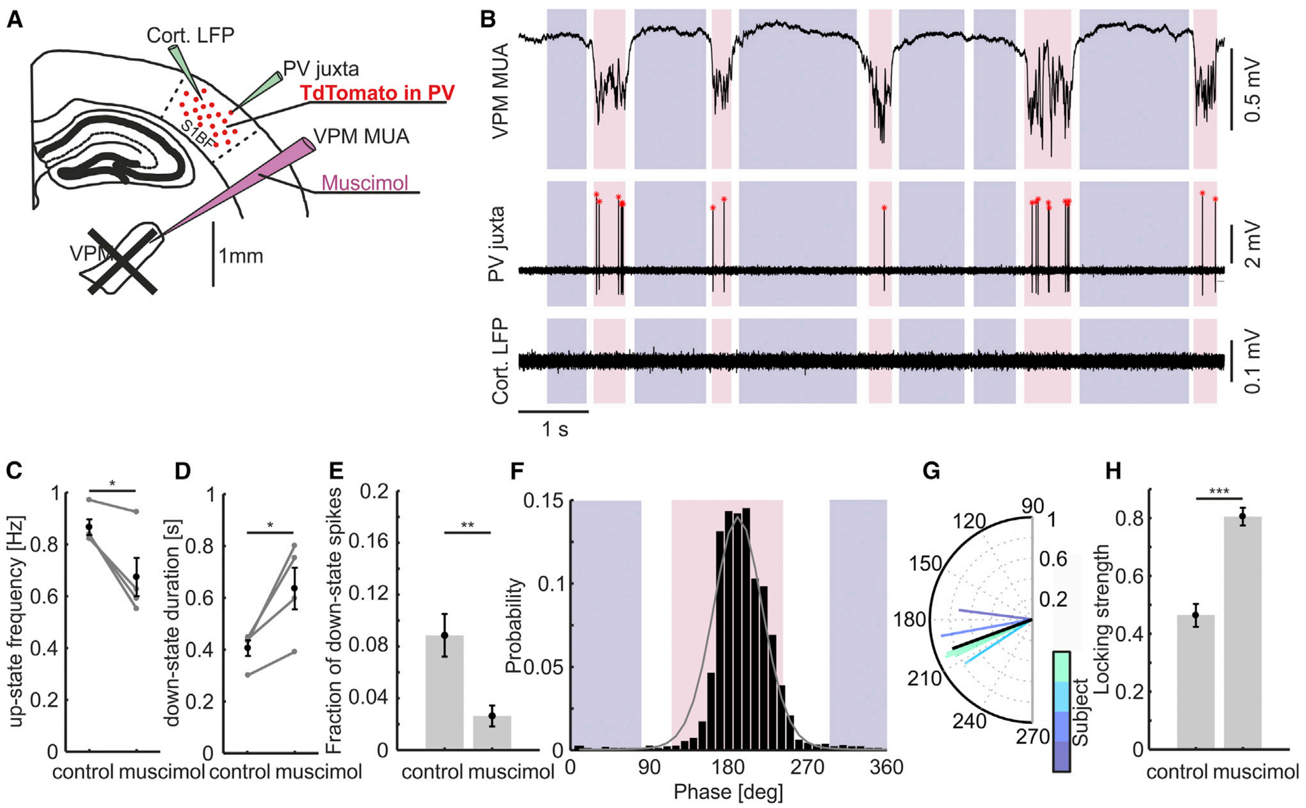

Figure 3. Thalamic Inactivation Significantly Reduces PV Spiking during Cortical Down States

(A) Schematic of the experimental configuration. Muscimol is injected in the thalamus while measuring cortical LFP, thalamic MUA, and one cortical PV interneuron.

(B) Representative traces of cortical LFP (top), juxtasomal recording from a PV interneuron (middle), and thalamic MUA (bottom) during up and down states after muscimol injection in the thalamus.

(C and D) Up-state frequency (C) and average down-state duration (D) under control conditions and after muscimol injection in the thalamus (paired Student's t test p = 0.0387 for C and p = 0.0367 for D; n = 4 animals).

(E) Fraction of PV spikes recorded during down states under control conditions and after muscimol injection in the thalamus (p = 6.24E−3, Student's t test; control, n = 8 cells; muscimol, n = 7 cells from 4 animals).

(F) Phase of firing distribution of one representative PV interneuron after muscimol injection in the thalamus. Pink and purple indicate the global range of up- and down-state phases after muscimol application, respectively. The gray line reports the corresponding von Mises fit ($\mu = 191°$ and $\kappa = 4.30$).

(G) Circular plot of the mean phase of firing of PV interneurons after muscimol application in the thalamus (n = 7 cells from 4 animals).

(H) Locking strength under control conditions and after muscimol injection (p = 1.25E−5, Student's t test; control, n = 8; muscimol, n = 7 from 4 animals).

See also Figure S3.

see Figure S3G legend). In contrast, the locking strength markedly increased upon thalamic inactivation (Figures 3F–3H), in agreement with the decreased occurrence of down-state spikes and increased probability of observing spikes in the up state. The phase of firing distribution for PV spikes during the up and down state was not changed after muscimol application with respect to control conditions (Figures S3H and S3I).

Down-to-Up Transitions Induced by Optogenetic Inhibition of PV Cells Show Increased Latency upon Thalamic Inactivation

Previous work demonstrated that the firing of PV interneurons during down states prolongs down-state durations [22]. More specifically, decreasing the probability of PV spiking in down states through optogenetic inhibitory manipulation reliably triggered short latency down-to-up-state transitions [22]. Here, we reasoned that, if the thalamus drives PV firing during down states, thalamic inactivation should prolong the latency of optogenetically induced down-to-up-state transitions. To test this hypothesis, we expressed the inhibitory opsin archaerhodopsin (Arch) [26] in PV cells while recording the LFP in S1bf before and after application of muscimol in the thalamus (Figure 4A). We confirmed that optical inhibition of PV cells during down states facilitated the up-state generation, resulting in a decrease of the latencies of down-to-up-state transitions compared to expected spontaneous transitions (latency: 0.11 ± 0.02 s for optically evoked transitions versus 0.32 ± 0.04 s for spontaneous transitions; n = 7 animals; p = 1.1E−3; paired Student's t test). Importantly, we found that pharmacological inhibition of the thalamus significantly prolonged the latencies of PV-inhibition-evoked down-to-up-state transitions when compared to PV-inhibition-evoked down-to-up-state transitions under control conditions (latency: 0.25 ± 0.4 s after muscimol application versus 0.11 ± 0.02 s under control conditions; n = 7 animals;

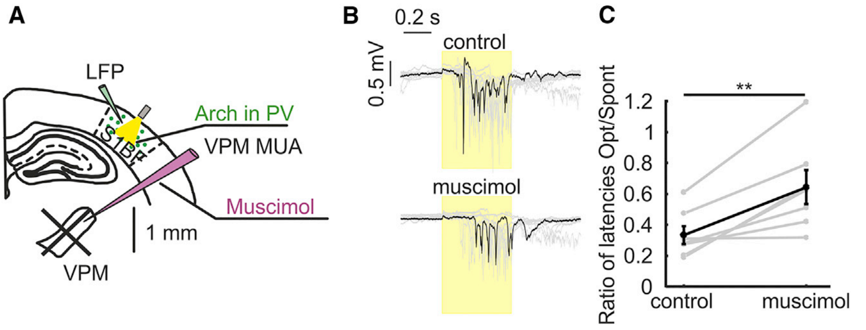

Figure 4. Increased Latency of Optogenetic-Evoked Down-to-Up Transitions upon Thalamic Inactivation

(A) Schematic of the experimental configuration. The inhibitory opsin Arch was expressed in PV cells located in S1bf (green circles). Cortical LFP was recorded via a glass electrode. Thalamic inhibition was achieved through local injection of muscimol.

(B) Representative cortical LFP traces under control conditions (top) and after muscimol injection in the thalamus (bottom), showing the effect of optogenetic inhibition of cortical PV interneurons (yellow bar).

(C) Ratio between light-evoked down-to-up transition latencies and spontaneous down-to-up transition latencies under control conditions and after muscimol application (n = 7 animals; p = 7.8E−3; Wilcoxon signed rank test).

See also Figure S4.

p = 1.7E−3; paired Student's t test; Figures 4B, 4C, and S4C). Moreover, the latency of optogenetically triggered down-to-up-state transitions was more similar to the latency of spontaneous down-to-up-state transitions upon thalamic inactivation, as demonstrated by the increase in the ratio between the latency of light-evoked transitions and the latency of spontaneous transitions after muscimol injection (Figure 4C). We confirmed the statistical significance of those results using a linear mixed effects model (see STAR Methods). We found that thalamic inactivation influenced the effect that the optogenetic manipulation had on up and down-state dynamics (p = 4E−3; type II Wald chi square tests for the interaction term between pharmacology and optogenetics).

Altogether, the results of these combined optogenetic and pharmacological experiments support the role of the thalamus in driving cortical PV interneuron spiking during down states.

DISCUSSION

Slow oscillations are the main oscillatory activity in the electroencephalogram (EEG), and they have long been considered of cortical origin. However, slow oscillations occur outside the cortex. For example, coordinated activity of cortical and thalamic networks is fundamental for their full expression [4, 27–30]. Early work [8] showed that thalamic neurons are active during slow oscillations. Moreover, thalamic spikes precede the initiation of an up state in the cortex [4, 21, 31, 32] and isolated thalamic slices displayed up and down states [33, 34]. Thalamic de-afferentation of the cortex significantly disrupted slow oscillations by reducing their frequency [25, 35]. Moreover, thalamic optogenetic stimulation reliably triggers up states and potentiates the EEG slow rhythms [4, 25, 36]. Sensory [10, 37, 38] or electrical thalamic [39] stimuli also triggered the generation of up states and slow oscillations in the cortex, suggesting that strong excitatory drive from the thalamus increases excitability in the cortex, ultimately favoring the appearance of a cortical up state.

Cortical inhibition is active during up and down states [40, 41], and inhibitory conductances have been shown to vary over the course of the up state [40, 42]. This inhibitory activity modulates up- and down-state transitions. Indeed, pharmacological blockade of the $GABA_A$ receptors [43] shortened up-state duration and pharmacological antagonism of the $GABA_B$ receptor prolonged it [44, 45]. A recent study combined cell-specific optogenetic manipulation targeted to cortical interneurons, including PV cells, with patch-clamp recordings *in vivo* to demonstrate that PV cell spiking controls both up-to-down and down-to-up-state transitions [22]. Specifically, PV cells were observed to fire during cortical down states and their spiking activity was associated with the prolongation of the down-state duration. Optogenetic inhibition of PV cells during cortical down states triggered a swift down-to-up-state transition, demonstrating that spiking of PV cells prolongs the duration of cortical down states [22]. However, what drives PV interneurons to fire during cortical down states remains unknown.

In this study, we provide a series of experimental evidences demonstrating that the thalamus contributes to drive PV cells to spike during cortical down states. Specifically, (1) the thalamic VPM nucleus is less phase locked to the up state of the slow oscillation compared to the S1bf and it is active during about half of cortical down states, (2) cortical down states have longer duration when the VPM is active compared to when the VPM is silent, (3) cortical down states, which display firing of PV cells, were associated with increased VPM activity, (4) the spike-triggered average of VPM activity based on the timing of PV spikes in down state showed increased VPM firing preceding the PV spike, (5) pharmacological blockade of the thalamus decreased PV spiking during down states, and (6) thalamic inactivation prolonged the latency of optogenetically induced down-to-up-state transitions. Remarkably, the involvement of the thalamus in driving interneurons was supported by non-perturbative experiments, in which we measured the spike activity of PV cells using two-photon guided juxtasomal electrophysiological recordings while recording network dynamics with the LFP and MUA, and pharmacological or optogenetic perturbative experiments, in which we modulated the firing of specific brain regions or specific cell types while recording network activity with electrophysiology.

In drawing these conclusions, it is important to underline that our results were obtained in anesthetized mice. Although recent studies investigating the role of specific cell types in the regulation of slow oscillation and up and down-state transitions found similar results in anesthetized and non-anesthetized mice [22, 46], future work will be needed to extend the findings presented in this study to sleeping animals [47].

VPM cells innervate both cortical principal neurons and GABAergic cells [48, 49], including PV interneurons [50–52]. If

VPM thalamic neurons fire during down states, they depolarize both cortical excitatory neurons and PV cells. However, a large body of previous work demonstrated that the firing of principal cells is largely silenced during cortical down states *in vivo* [3, 53, 54] (but see also [55–57]). In contrast, PV interneurons display spiking activity during these cortical silent states *in vivo* [21, 22, 56]. Thus, one possibility is that VPM directly drives PV cells during down states. This hypothesis implies that thalamo-cortical synapses are more efficient in driving suprathreshold response in PV cells compared to principal neurons, in agreement with previous work [58, 59]. It is important to underline, however, that regardless of whether the effect is direct (VPM → PV cell) or indirect (VPM → principal cell → PV cell), our data show that the VPM significantly drives PV cells to spike during cortical down states.

It is also important to note that down-to-up transitions in the cortex spontaneously occurred under conditions of largely reduced (but not suppressed; see Figure 3E) firing of PV cells during down states, as after muscimol application in the thalamus (Figure 3B). This suggests that PV interneurons are not necessary for the down-to-up transition to occur. Rather, through their spiking activity during down states, they prolong the cortical down-state duration and control the timing of the down-to-up transition, in agreement with previous observations [22].

In the framework of what was discussed above, it must be underlined that slow oscillations also occur in isolated cortical slices *in vitro* in the absence of the thalamus [42, 55] and *in vivo* following thalamic inactivation [25, 35] (see Figure 3) or cortical de-afferentation [35, 60]. Thus, the thalamic drive of cortical interneurons that we describe in this study is just one mechanism controlling state changes, which will likely be integrated with other fundamental cortical processes during spontaneous up and down-state transitions. Among these processes, activity-dependent K^+ currents, in particular Ca^{2+}-dependent K^+ current [61] and Na^+-dependent K^+ currents [62], are thought to play a critical role in cortical slow oscillations [55] powerfully contributing to the termination of the up state and generating prolonged hyperpolarization (down state) [63, 64]. Other cortical mechanisms controlling up- and down-state transitions may, for example, involve changes in the concentration of extracellular Ca^{2+} [65].

In our study, we restricted the investigation to PV cells. However, SOM interneurons have been shown to fire during the down state [22, 56, 66], and their perturbation has been demonstrated to profoundly modulate up- and down-state transitions [22] and slow oscillations [67]. Given that SOM interneurons, as well as other types of cortical GABAergic cells, also receive direct excitatory input from thalamo-cortical cells [51, 68], the thalamus may modulate state transitions through the active recruitment of interneuronal populations other than PV cells.

Our results demonstrate that thalamic spontaneous activity can have an overall inhibitory effect on cortical network dynamics during slow oscillations. This is in line with previous observations [69] suggesting that thalamo-cortical inputs to the neocortex may synchronize inhibitory interneurons, thus contributing to synchronous up-to-down-state transitions via feedforward inhibition. Although it is clear that strong thalamic activity induced by sensory [37], electrical [39], or optogenetic [25, 36] stimulation enhances excitation in the cortex, ultimately resulting in the generation of an up-state transition, the data presented in this study demonstrate that moderate level of spontaneous thalamic activity results in inhibitory (rather than excitatory) effects on cortical networks (i.e., prolongation of down-state duration). On the basis of the evidences presented in this study and of those described in the literature, we here propose a unifying view in which the thalamic firing level is seen as a control signal for cortical state transitions. According to this hypothesis, low spontaneous thalamic firing during cortical down states results in prevailing inhibitory effect onto cortex, e.g., prolonging silent cortical states through the action of PV interneurons. In contrast, progressively higher thalamic firing results in cortical networks excitation, thus promoting down-to-up-state transitions. This hypothesis is in agreement with the observation that thalamo-cortical connections form direct synapses onto cortical interneurons [70, 71] and that these connections are stronger and more effective than thalamo-cortical connections onto pyramidal excitatory neurons [58, 59, 68, 72].

One intriguing question is whether our findings in the somatosensory thalamo-cortical loop can be extended to other thalamo-cortical circuits, including those involving non-sensory areas (e.g., the centromedial thalamus [CMT] and the cingulate cortex), which are involved in the control up and down-state transitions [4]. Same similarities between different thalamo-cortical loops can be observed. For example, VPM thalamo-cortical neurons fire in advance of S1 cortical cells at up state start [21], and the firing of CMT neurons similarly precedes up-state generation in the cingulate cortex [4]. However, the spiking activity of CMT and VB neurons is differentially modulated during sleep-wake cycle, and optogenetic manipulation of CMT and VB neurons have different effects on sleep-wake transitions [4]. Thus, it is conceivable whether our findings can be extended to non-somatosensory thalamo-cortical circuits.

In summary, we demonstrated that the thalamus drives PV interneurons to fire during cortical down states, exerting an inhibitory control of cortical S1 network dynamics during down states. Given the role of up- and down-state transitions in memory consolidation [9], synaptic plasticity [15, 16], and the modulation of sensory responses [10, 12], these findings highlight the importance of precisely dissecting out the complex circuit mechanisms underlying the function of the thalamo-cortical system.

STAR★METHODS

Detailed methods are provided in the online version of this paper and include the following:

- KEY RESOURCES TABLE
- CONTACT FOR REAGENT AND RESOURCE SHARING
- EXPERIMENTAL MODEL AND SUBJECT DETAILS
- METHOD DETAILS
  - Viral Injections
  - Electrophysiology
  - Intrinsic optical imaging
  - Whisker stimulation
  - Pharmacology and optogenetics
- QUANTIFICATION AND STATISTICAL ANALYSIS
  - Data selection
  - Up- and down-state detection from LFP signal
  - Spike detection

  - ○ Phase locking analysis between cortical PV interneurons / thalamic activity and LFP
  - ○ Spike-triggered thalamic instantaneous firing rate
  - ○ Evoked down-to-up transition latency
  - ○ Statistics
- DATA AND SOFTWARE AVAILABILITY

SUPPLEMENTAL INFORMATION

Supplemental Information can be found online at https://doi.org/10.1016/j.cub.2019.04.007.

ACKNOWLEDGMENTS

This work was supported by ERC (NEURO-PATTERNS), NIH (U01 NS090576 and U19 NS107464), FP7 (DESIRE), and, in part, by Flag-Era JTC Human Brain Project (SLOW-DYN).

AUTHOR CONTRIBUTIONS

S.Z. performed experiments. V.P., P.L.d.L.R., S.Z., and S.P. performed analysis. T.F. conceived and coordinated the project. V.P., P.L.d.L.R., S.Z., S.P., and T.F. wrote the manuscript.

DECLARATION OF INTERESTS

The authors declare no competing interests.

Received: December 21, 2018
Revised: March 8, 2019
Accepted: April 3, 2019
Published: April 25, 2019

REFERENCES

1. Steriade, M., Contreras, D., Curró Dossi, R., and Nuñez, A. (1993). The slow (< 1 Hz) oscillation in reticular thalamic and thalamocortical neurons: scenario of sleep rhythm generation in interacting thalamic and neocortical networks. J. Neurosci. *13*, 3284–3299.
2. Steriade, M., Nuñez, A., and Amzica, F. (1993). A novel slow (< 1 Hz) oscillation of neocortical neurons in vivo: depolarizing and hyperpolarizing components. J. Neurosci. *13*, 3252–3265.
3. Steriade, M., Nuñez, A., and Amzica, F. (1993). Intracellular analysis of relations between the slow (< 1 Hz) neocortical oscillation and other sleep rhythms of the electroencephalogram. J. Neurosci. *13*, 3266–3283.
4. Gent, T.C., Bandarabadi, M., Herrera, C.G., and Adamantidis, A.R. (2018). Thalamic dual control of sleep and wakefulness. Nat. Neurosci. *21*, 974–984.
5. Massimini, M., Ferrarelli, F., Esser, S.K., Riedner, B.A., Huber, R., Murphy, M., Peterson, M.J., and Tononi, G. (2007). Triggering sleep slow waves by transcranial magnetic stimulation. Proc. Natl. Acad. Sci. USA *104*, 8496–8501.
6. Mukamel, E.A., Pirondini, E., Babadi, B., Wong, K.F.K., Pierce, E.T., Harrell, P.G., Walsh, J.L., Salazar-Gomez, A.F., Cash, S.S., Eskandar, E.N., et al. (2014). A transition in brain state during propofol-induced unconsciousness. J. Neurosci. *34*, 839–845.
7. Saleem, A.B., Chadderton, P., Apergis-Schoute, J., Harris, K.D., and Schultz, S.R. (2010). Methods for predicting cortical UP and DOWN states from the phase of deep layer local field potentials. J. Comput. Neurosci. *29*, 49–62.
8. Contreras, D., and Steriade, M. (1995). Cellular basis of EEG slow rhythms: a study of dynamic corticothalamic relationships. J. Neurosci. *15*, 604–622.
9. Marshall, L., Helgadóttir, H., Mölle, M., and Born, J. (2006). Boosting slow oscillations during sleep potentiates memory. Nature *444*, 610–613.
10. Petersen, C.C.H., Hahn, T.T.G., Mehta, M., Grinvald, A., and Sakmann, B. (2003). Interaction of sensory responses with spontaneous depolarization in layer 2/3 barrel cortex. Proc. Natl. Acad. Sci. USA *100*, 13638–13643.
11. Crochet, S., Chauvette, S., Boucetta, S., and Timofeev, I. (2005). Modulation of synaptic transmission in neocortex by network activities. Eur. J. Neurosci. *21*, 1030–1044.
12. Haider, B., Duque, A., Hasenstaub, A.R., Yu, Y., and McCormick, D.A. (2007). Enhancement of visual responsiveness by spontaneous local network activity in vivo. J. Neurophysiol. *97*, 4186–4202.
13. Reig, R., Zerlaut, Y., Vergara, R., Destexhe, A., and Sanchez-Vives, M.V. (2015). Gain modulation of synaptic inputs by network state in auditory cortex in vivo. J. Neurosci. *35*, 2689–2702.
14. Tononi, G., and Cirelli, C. (2014). Sleep and the price of plasticity: from synaptic and cellular homeostasis to memory consolidation and integration. Neuron *81*, 12–34.
15. Vyazovskiy, V.V., Cirelli, C., Pfister-Genskow, M., Faraguna, U., and Tononi, G. (2008). Molecular and electrophysiological evidence for net synaptic potentiation in wake and depression in sleep. Nat. Neurosci. *11*, 200–208.
16. Chauvette, S., Seigneur, J., and Timofeev, I. (2012). Sleep oscillations in the thalamocortical system induce long-term neuronal plasticity. Neuron *75*, 1105–1113.
17. Timofeev, I., and Chauvette, S. (2017). Sleep slow oscillation and plasticity. Curr. Opin. Neurobiol. *44*, 116–126.
18. Crunelli, V., Lőrincz, M.L., Connelly, W.M., David, F., Hughes, S.W., Lambert, R.C., Leresche, N., and Errington, A.C. (2018). Dual function of thalamic low-vigilance state oscillations: rhythm-regulation and plasticity. Nat. Rev. Neurosci. *19*, 107–118.
19. Timofeev, I., Grenier, F., and Steriade, M. (2001). Disfacilitation and active inhibition in the neocortex during the natural sleep-wake cycle: an intracellular study. Proc. Natl. Acad. Sci. USA *98*, 1924–1929.
20. Chauvette, S., Crochet, S., Volgushev, M., and Timofeev, I. (2011). Properties of slow oscillation during slow-wave sleep and anesthesia in cats. J. Neurosci. *31*, 14998–15008.
21. Ushimaru, M., and Kawaguchi, Y. (2015). Temporal structure of neuronal activity among cortical neuron subtypes during slow oscillations in anesthetized rats. J. Neurosci. *35*, 11988–12001.
22. Zucca, S., D'Urso, G., Pasquale, V., Vecchia, D., Pica, G., Bovetti, S., Moretti, C., Varani, S., Molano-Mazón, M., Chiappalone, M., et al. (2017). An inhibitory gate for state transition in cortex. eLife *6*, 1–31.
23. Mukovski, M., Chauvette, S., Timofeev, I., and Volgushev, M. (2007). Detection of active and silent states in neocortical neurons from the field potential signal during slow-wave sleep. Cereb. Cortex *17*, 400–414.
24. Panzeri, S., Harvey, C.D., Piasini, E., Latham, P.E., and Fellin, T. (2017). Cracking the neural code for sensory perception by combining statistics, intervention, and behavior. Neuron *93*, 491–507.
25. David, F., Schmiedt, J.T., Taylor, H.L., Orban, G., Di Giovanni, G., Uebele, V.N., Renger, J.J., Lambert, R.C., Leresche, N., and Crunelli, V. (2013). Essential thalamic contribution to slow waves of natural sleep. J. Neurosci. *33*, 19599–19610.
26. Chow, B.Y., Han, X., Dobry, A.S., Qian, X., Chuong, A.S., Li, M., Henninger, M.A., Belfort, G.M., Lin, Y., Monahan, P.E., and Boyden, E.S. (2010). High-performance genetically targetable optical neural silencing by light-driven proton pumps. Nature *463*, 98–102.
27. Crunelli, V., and Hughes, S.W. (2010). The slow (<1 Hz) rhythm of non-REM sleep: a dialogue between three cardinal oscillators. Nat. Neurosci. *13*, 9–17.
28. Crunelli, V., Errington, A.C., Hughes, S.W., and Tóth, T.I. (2011). The thalamic low-threshold Ca^{2+} potential: a key determinant of the local and global dynamics of the slow (<1 Hz) sleep oscillation in thalamocortical networks. Philos. Trans. A Math. Phys. Eng. Sci. *369*, 3820–3839.
29. Crunelli, V., David, F., Lőrincz, M.L., and Hughes, S.W. (2015). The thalamocortical network as a single slow wave-generating unit. Curr. Opin. Neurobiol. *31*, 72–80.

30. Gent, T.C., Bassetti, C., and Adamantidis, A.R. (2018). Sleep-wake control and the thalamus. Curr. Opin. Neurobiol. *52*, 188–197.

31. Slézia, A., Hangya, B., Ulbert, I., and Acsády, L. (2011). Phase advancement and nucleus-specific timing of thalamocortical activity during slow cortical oscillation. J. Neurosci. *31*, 607–617.

32. Sheroziya, M., and Timofeev, I. (2014). Global intracellular slow-wave dynamics of the thalamocortical system. J. Neurosci. *34*, 8875–8893.

33. Hughes, S.W., Cope, D.W., Blethyn, K.L., and Crunelli, V. (2002). Cellular mechanisms of the slow (<1 Hz) oscillation in thalamocortical neurons in vitro. Neuron *33*, 947–958.

34. Blethyn, K.L., Hughes, S.W., Tóth, T.I., Cope, D.W., and Crunelli, V. (2006). Neuronal basis of the slow (<1 Hz) oscillation in neurons of the nucleus reticularis thalami in vitro. J. Neurosci. *26*, 2474–2486.

35. Lemieux, M., Chen, J.-Y., Lonjers, P., Bazhenov, M., and Timofeev, I. (2014). The impact of cortical deafferentation on the neocortical slow oscillation. J. Neurosci. *34*, 5689–5703.

36. Poulet, J.F.A., Fernandez, L.M.J., Crochet, S., and Petersen, C.C.H. (2012). Thalamic control of cortical states. Nat. Neurosci. *15*, 370–372.

37. Civillico, E.F., and Contreras, D. (2012). Spatiotemporal properties of sensory responses in vivo are strongly dependent on network context. Front. Syst. Neurosci. *6*, 25.

38. Ngo, H.-V.V., Martinetz, T., Born, J., and Mölle, M. (2013). Auditory closed-loop stimulation of the sleep slow oscillation enhances memory. Neuron *78*, 545–553.

39. MacLean, J.N., Watson, B.O., Aaron, G.B., and Yuste, R. (2005). Internal dynamics determine the cortical response to thalamic stimulation. Neuron *48*, 811–823.

40. Neske, G.T. (2016). The slow oscillation in cortical and thalamic networks: mechanisms and functions. Front. Neural Circuits *9*, 88.

41. Puig, M.V., Ushimaru, M., and Kawaguchi, Y. (2008). Two distinct activity patterns of fast-spiking interneurons during neocortical UP states. Proc. Natl. Acad. Sci. USA *105*, 8428–8433.

42. Shu, Y., Hasenstaub, A., and McCormick, D.A. (2003). Turning on and off recurrent balanced cortical activity. Nature *423*, 288–293.

43. Sanchez-Vives, M.V., Mattia, M., Compte, A., Perez-Zabalza, M., Winograd, M., Descalzo, V.F., and Reig, R. (2010). Inhibitory modulation of cortical up states. J. Neurophysiol. *104*, 1314–1324.

44. Mann, E.O., Kohl, M.M., and Paulsen, O. (2009). Distinct roles of GABA(A) and GABA(B) receptors in balancing and terminating persistent cortical activity. J. Neurosci. *29*, 7513–7518.

45. Craig, M.T., and McBain, C.J. (2014). The emerging role of GABAB receptors as regulators of network dynamics: fast actions from a 'slow' receptor? Curr. Opin. Neurobiol. *26*, 15–21.

46. Beltramo, R., D'Urso, G., Dal Maschio, M., Farisello, P., Bovetti, S., Clovis, Y., Lassi, G., Tucci, V., De Pietri Tonelli, D., and Fellin, T. (2013). Layer-specific excitatory circuits differentially control recurrent network dynamics in the neocortex. Nat. Neurosci. *16*, 227–234.

47. Urbain, N., Fourcaud-Trocmé, N., Laheux, S., Salin, P.A., and Gentet, L.J. (2019). Brain-State-Dependent Modulation of Neuronal Firing and Membrane Potential Dynamics in the Somatosensory Thalamus during Natural Sleep. Cell Rep. *26*, 1443–1457.

48. Brecht, M. (2007). Barrel cortex and whisker-mediated behaviors. Curr. Opin. Neurobiol. *17*, 408–416.

49. Feldmeyer, D., Brecht, M., Helmchen, F., Petersen, C.C.H., Poulet, J.F.A., Staiger, J.F., Luhmann, H.J., and Schwarz, C. (2013). Barrel cortex function. Prog. Neurobiol. *103*, 3–27.

50. Ji, X.Y., Zingg, B., Mesik, L., Xiao, Z., Zhang, L.I., and Tao, H.W. (2016). Thalamocortical innervation pattern in mouse auditory and visual cortex: laminar and cell-type specificity. Cereb. Cortex *26*, 2612–2625.

51. Tan, Z., Hu, H., Huang, Z.J., and Agmon, A. (2008). Robust but delayed thalamocortical activation of dendritic-targeting inhibitory interneurons. Proc. Natl. Acad. Sci. USA *105*, 2187–2192.

52. Wall, N.R., De La Parra, M., Sorokin, J.M., Taniguchi, H., Huang, Z.J., and Callaway, E.M. (2016). Brain-wide maps of synaptic input to cortical interneurons. J. Neurosci. *36*, 4000–4009.

53. Sakata, S., and Harris, K.D. (2009). Laminar structure of spontaneous and sensory-evoked population activity in auditory cortex. Neuron *64*, 404–418.

54. Chauvette, S., Volgushev, M., and Timofeev, I. (2010). Origin of active states in local neocortical networks during slow sleep oscillation. Cereb. Cortex *20*, 2660–2674.

55. Sanchez-Vives, M.V., and McCormick, D.A. (2000). Cellular and network mechanisms of rhythmic recurrent activity in neocortex. Nat. Neurosci. *3*, 1027–1034.

56. Fanselow, E.E., and Connors, B.W. (2010). The roles of somatostatin-expressing (GIN) and fast-spiking inhibitory interneurons in UP-DOWN states of mouse neocortex. J. Neurophysiol. *104*, 596–606.

57. Senzai, Y., Fernandez-Ruiz, A., and Buzsáki, G. (2019). Layer-specific physiological features and interlaminar interactions in the primary visual cortex of the mouse. Neuron *101*, 500–513.e5.

58. Cruikshank, S.J., Lewis, T.J., and Connors, B.W. (2007). Synaptic basis for intense thalamocortical activation of feedforward inhibitory cells in neocortex. Nat. Neurosci. *10*, 462–468.

59. Hull, C., Isaacson, J.S., and Scanziani, M. (2009). Postsynaptic mechanisms govern the differential excitation of cortical neurons by thalamic inputs. J. Neurosci. *29*, 9127–9136.

60. Timofeev, I., Grenier, F., Bazhenov, M., Sejnowski, T.J., and Steriade, M. (2000). Origin of slow cortical oscillations in deafferented cortical slabs. Cereb. Cortex *10*, 1185–1199.

61. Schwindt, P.C., Spain, W.J., and Crill, W.E. (1992). Calcium-dependent potassium currents in neurons from cat sensorimotor cortex. J. Neurophysiol. *67*, 216–226.

62. Schwindt, P.C., Spain, W.J., and Crill, W.E. (1989). Long-lasting reduction of excitability by a sodium-dependent potassium current in cat neocortical neurons. J. Neurophysiol. *61*, 233–244.

63. Compte, A., Sanchez-Vives, M.V., McCormick, D.A., and Wang, X.-J. (2003). Cellular and network mechanisms of slow oscillatory activity (<1 Hz) and wave propagations in a cortical network model. J. Neurophysiol. *89*, 2707–2725.

64. Hill, S., and Tononi, G. (2005). Modeling sleep and wakefulness in the thalamocortical system. J. Neurophysiol. *93*, 1671–1698.

65. Massimini, M., and Amzica, F. (2001). Extracellular calcium fluctuations and intracellular potentials in the cortex during the slow sleep oscillation. J. Neurophysiol. *85*, 1346–1350.

66. Tahvildari, B., Wölfel, M., Duque, A., and McCormick, D.A. (2012). Selective functional interactions between excitatory and inhibitory cortical neurons and differential contribution to persistent activity of the slow oscillation. J. Neurosci. *32*, 12165–12179.

67. Funk, C.M., Peelman, K., Bellesi, M., Marshall, W., Cirelli, C., and Tononi, G. (2017). Role of somatostatin-positive cortical interneurons in the generation of sleep slow waves. J. Neurosci. *37*, 9132–9148.

68. Cruikshank, S.J., Urabe, H., Nurmikko, A.V., and Connors, B.W. (2010). Pathway-specific feedforward circuits between thalamus and neocortex revealed by selective optical stimulation of axons. Neuron *65*, 230–245.

69. Lemieux, M., Chauvette, S., and Timofeev, I. (2015). Neocortical inhibitory activities and long-range afferents contribute to the synchronous onset of silent states of the neocortical slow oscillation. J. Neurophysiol. *113*, 768–779.

70. Swadlow, H.A. (1995). Influence of VPM afferents on putative inhibitory interneurons in S1 of the awake rabbit: evidence from cross-correlation, microstimulation, and latencies to peripheral sensory stimulation. J. Neurophysiol. *73*, 1584–1599.

71. Feldmeyer, D., Qi, G., Emmenegger, V., and Staiger, J.F. (2018). Inhibitory interneurons and their circuit motifs in the many layers of the barrel cortex. Neuroscience *368*, 132–151.

72. Bagnall, M.W., Hull, C., Bushong, E.A., Ellisman, M.H., and Scanziani, M. (2011). Multiple clusters of release sites formed by individual thalamic afferents onto cortical interneurons ensure reliable transmission. Neuron *71*, 180–194.

73. Maccione, A., Gandolfo, M., Massobrio, P., Novellino, A., Martinoia, S., and Chiappalone, M. (2009). A novel algorithm for precise identification of spikes in extracellularly recorded neuronal signals. J. Neurosci. Methods *177*, 241–249.

74. Forli, A., Vecchia, D., Binini, N., Succol, F., Bovetti, S., Moretti, C., Nespoli, F., Mahn, M., Baker, C.A., Bolton, M.M., et al. (2018). Two-photon bidirectional control and imaging of neuronal excitability with high spatial resolution in vivo. Cell Rep. *22*, 3087–3098.

75. De Stasi, A.M., Farisello, P., Marcon, I., Cavallari, S., Forli, A., Vecchia, D., Losi, G., Mantegazza, M., Panzeri, S., Carmignoto, G., et al. (2016). Unaltered network activity and interneuronal firing during spontaneous cortical dynamics in vivo in a mouse model of severe myoclonic epilepsy of infancy. Cereb. Cortex *26*, 1778–1794.

76. Bovetti, S., Moretti, C., Zucca, S., Dal Maschio, M., Bonifazi, P., and Fellin, T. (2017). Simultaneous high-speed imaging and optogenetic inhibition in the intact mouse brain. Sci. Rep. *7*, 40041.

77. Harrison, T.C., Sigler, A., and Murphy, T.H. (2009). Simple and cost-effective hardware and software for functional brain mapping using intrinsic optical signal imaging. J. Neurosci. Methods *182*, 211–218.

78. Li, C.Y., Poo, M.M., and Dan, Y. (2009). Burst spiking of a single cortical neuron modifies global brain state. Science *324*, 643–646.

79. Barbaresi, P., Spreafico, R., Frassoni, C., and Rustioni, A. (1986). GABAergic neurons are present in the dorsal column nuclei but not in the ventroposterior complex of rats. Brain Res. *382*, 305–326.

80. Arcelli, P., Frassoni, C., Regondi, M.C., De Biasi, S., and Spreafico, R. (1997). GABAergic neurons in mammalian thalamus: a marker of thalamic complexity? Brain Res. Bull. *42*, 27–37.

81. Barthó, P., Slézia, A., Mátyás, F., Faradzs-Zade, L., Ulbert, I., Harris, K.D., and Acsády, L. (2014). Ongoing network state controls the length of sleep spindles via inhibitory activity. Neuron *82*, 1367–1379.

82. Eschenko, O., Magri, C., Panzeri, S., and Sara, S.J. (2012). Noradrenergic neurons of the locus coeruleus are phase locked to cortical up-down states during sleep. Cereb. Cortex *22*, 426–435.

83. Siapas, A.G., Lubenov, E.V., and Wilson, M.A. (2005). Prefrontal phase locking to hippocampal theta oscillations. Neuron *46*, 141–151.

84. Montemurro, M.A., Rasch, M.J., Murayama, Y., Logothetis, N.K., and Panzeri, S. (2008). Phase-of-firing coding of natural visual stimuli in primary visual cortex. Curr. Biol. *18*, 375–380.

85. Pinheiro, J.C., and Bates, D.M. (2000). Linear mixed-effects models: basic concepts and examples. Mixed-Effects Models in S and S-PLUS (Springer-Verlag), pp. 3–56.

86. Fox, J. (1997). Applied Regression Analysis, Linear Models, and Related Methods (SAGE Publications).

STAR★METHODS

KEY RESOURCES TABLE

REAGENT or RESOURCE	SOURCE	IDENTIFIER
Bacterial and Virus Strains		
AAV1.CBA.Flex.Arch-GFP.WPRE.SV40	Penn Vector Core	RRID:Addgene_22222; Addgene viral prep # 22222-AAV1
Chemicals, Peptides, and Recombinant Proteins		
Muscimol, BODIPY TMR-X Conjugate	Thermo Fisher Scientific	Cat#:M23400
Urethane, > 99%	Sigma-Aldrich	Cat#:U2500; CAS: 51-79-6
Alexa 488 Fluor Hydrazide	Thermo Fisher Scientific	Cat#:A10436
Deposited Data		
Raw electrophysiological recordings (datasets 1 & 2)	Zenodo	DOI: https://www.doi.org/10.5281/zenodo.2609157
Experimental Models: Organisms/Strains		
Mouse: B6;129S6-*Gt(ROSA)26Sor*$^{tm14(CAG-TdTomato)Hze}$/*J*	Jackson Laboratory	RRID:IMSR_JAX:007908
Mouse: B6;129P2-*Pvalb*$^{tm1(cre)Arbr}$/*J*	Jackson Laboratory	RRID:IMSR_JAX:008069
Software and Algorithms		
MATLAB R2017a	Mathworks	RRID:SCR_001622; URL: http://www.mathworks.com/products/matlab/
Origin 2016 64bit	OriginLab	RRID:SCR_002815; URL: https://www.originlab.com/
RStudio	RStudio	RRID:SCR_000432; URL: http://www.rstudio.com/
Spike detection	See [73]	https://doi.org/10.1016/j.jneumeth.2008.09.026
Custom MATLAB code (up/down state detection, phase locking, spike-triggered IFR, evoked transitions latencies algorithms)	This paper; Zenodo	DOI: https://www.doi.org/10.5281/zenodo.2609157

CONTACT FOR REAGENT AND RESOURCE SHARING

Further information and requests for resources and reagents should be directed to and will be fulfilled by the Lead Contact, Tommaso Fellin (tommaso.fellin@iit.it).

EXPERIMENTAL MODEL AND SUBJECT DETAILS

Experimental procedures involving animals have been approved by the IIT Animal Welfare Body and by the Italian Ministry of Health (authorization # 34/2015-PR and 125/2012-B), in accordance with the National legislation (D.Lgs. 26/2014) and the European legislation (European Directive 2010/63/EU). The mouse lines B6;129S6-*Gt(ROSA)26Sor*$^{tm14(CAG-TdTomato)Hze}$/*J*, id #007908, (otherwise called TdTomato line) and B6;129P2-*Pvalb*$^{tm1(cre)Arbr}$/*J*, id #008069, (called PV-cre line) were purchased from the Jackson Laboratory (Bar Harbor, USA). The animals were housed in a 12:12 hr light-dark cycle in individually ventilated cages, with access to food and water *ad libitum*.

METHOD DETAILS

Viral Injections

The adeno-associated virus AAV1.CBA.Flex.Arch-GFP.WPRE.SV40 (Arch) was purchased from the University of Pennsylvania Viral Vector Core. PV-Cre transgenic mice (both males and females) were injected between postnatal day 0 (P0) and P2. Pups were anesthetized using hypothermia, placed on a custom-made stereotaxic apparatus and kept at approximately 4°C for the entire duration of

the surgery. A small skin incision was used to expose the skull and ~250 nL of viral suspension were injected using a micropipette at stereotaxic coordinates of 0 mm from bregma, 2 mm lateral of the sagittal sinus, and 0.25–0.3 mm depth. Following injection, the micropipette was held in place for 1–2 min before retraction. After pipette removal, the skin was sutured, and the pup was revitalized under an infrared heating lamp.

Electrophysiology

Electrophysiological recordings were performed at postnatal day P24-P28 for PV-cre and PV-cre x TdTomato mice. Urethane anesthesia (16.5%, 1.65 g/kg) was used to induce cortical spontaneous up- and down-states transitions. The body temperature was monitored using a rectal probe and maintained at 37°C with a heating pad. Oxygen saturation was controlled by a pulse oximeter (MouseOx, Starr Life Sciences, Oakmont, PA). The depth of anesthesia throughout the surgery and the experiments was controlled by monitoring respiration rate, heartbeat, eyelid reflex, vibrissae movements, reactions to tail and toe pinching. Simultaneous cortical LFP and two-photon-guided PV juxtasomal recordings (Figures 1 and 3) were performed as in [22] and centered on top of the barrel cortex. Briefly, a low resistance (0.3-0.6 MΩ) pipette, filled with HEPES-buffered artificial cerebrospinal solution (ACSF), was lowered at ~300 μm depth from the pial surface to monitor superficial LFP activity. Juxtasomal recordings were performed with a second high resistance (5-8 MΩ) pipette, filled with ACSF and 2 mM Alexa 488 Fluor (Thermo Fisher Scientific, Waltham, MA, USA) [74, 75]. Superficial (80-350 μm below the pial surface) PV interneurons were identified by imaging TdTomato fluorescence with an Ultima II laser scanning two-photon microscope (Bruker, Billerica, MA, former Prairie Technologies, Madison, WI, USA) coupled to a Chameleon Ultra II (Coherent Santa Clara, CA, λ_{exc} = 720 nm) [76]. In addition, a third craniotomy was performed at specific coordinates (AP: 3.8, ML: 1.7) which allows to selectively target the thalamic VPM nucleus by lowering a glass pipette.

For LFP recordings during optogenetic manipulation of PV interneurons (Figure 4), Arch expression was first controlled by looking at GFP fluorescence through the mouse skull under a fluorescence stereo microscope. The skull was thinned on top of the barrel cortex where Arch expression was higher. In a subset of experiments, intrinsic optical imaging (IOI) was then performed by stimulating a single whisker (usually B2 or C2). The selected whisker or neighboring ones (e.g., C1, C3 if C2 was used for IOI) were later used to control for whisker-evoked responses in the VPM. A small craniotomy (0.5 mm x 0.5 mm) was performed on top of the identified area and a low resistance (0.5-1 MOhm) glass pipette was lowered to collect the LFP signals from superficial layers (~350 μm from cortical surface). At the same time a second craniotomy was performed at the following coordinates (AP = 3.8, ML = 1.8) and a tilted glass capillary was placed at a depth of about 3.2-3.5 mm in order to target the VPM and to collect its multi-unit activity (MUA) signal.

In all the experiments the cortical surface was kept moist with ACSF. LFP signal displayed in Figures 1 and 3 was filtered in the bandwidth 0.1 – 1 kHz, amplified by an AM-amplifier (AM-system, Carlsborg, WA, USA) and digitized at 10 kHz with a Digidata 1440 (Axon Instruments, Union City, CA). MUA and juxtasomal signals were acquired using a Multiclamp 700B amplifier, filtered at 2.2 kHz and 6 kHz respectively, digitized at 10 kHz with a Digidata 1440. For experiments displayed in Figure 4, all the signals were filtered at 0.1 – 2 kHz, acquired using a Multiclamp 700B, and digitized at 50 kHz. All data were stored with pClamp 10 (Axon Instruments, Union City, CA, USA).

Intrinsic optical imaging

IOI was performed with a customized set-up. The skin was cut to expose the skull and the area above the primary somatosensory cortex was thinned. A single whisker (usually B2 or C2) was introduced in a glass capillary tube glued to a piezoelectric bender actuator (Physik Instrumente, Milan, IT), and then stimulated at 18 Hz for 1.1 s at intervals of 20 s for a total of 40 trials. Illumination upon the skull was done with red light (630 ± 10 nm) and a CCD camera (Hamamatsu, Milan, IT) was used to acquire time series images during whisker stimulation. The analysis of the images was performed with a custom MATLAB script based on [77]. The region that showed lower reflectance compared to baseline was used to identify the principal barrel corresponding to that whisker. Blood vessels were used as reference by acquiring an image under green light (546 ± 10 nm). Once the area of interest was identified by IOI, a small craniotomy (~1 mm^2) was performed over that region.

Whisker stimulation

To assess the position of pipettes used for recording and pharmacological application in the thalamic VPM nucleus, we stimulated the mouse whiskers with a single pulse of compressed air delivered through a glass capillary and, at the same time, we recorded the MUA activity in the VPM. The pulses were 20-ms long and presented at 0.25 Hz. We evaluated the responsiveness of the recorded region by detecting extracellular spikes as described before and calculating the spike rate in 100-ms windows before and after the onset of the stimulation (pre- and post-stimulation windows). Under control conditions, we found a significant increase in the spike rate after whisker stimulation with respect to the preceding time period (pre stimulation: 8.14 ± 2.62 spikes/s, post stimulation: 24.09 ± 3.10 spikes/s, mean ± s.e.m., n = 7 animals, p = 0.0074, paired Student's t test). After muscimol injection VPM was largely suppressed with no significant difference between activity preceding and following whisker stimulation (no detected spikes in pre-stimulation windows versus 2.38 ± 2.30 spikes/s in post-window, mean ± sem, n = 7 animals, p = 0.34, one-sample Student's t test). We computed the spike rate of the complete recordings averaged across animals during spontaneous activity, obtaining: 5.71 ± 1.65 spikes/s under control condition and 0.02 ± 0.01 spikes/s after muscimol injection.

Pharmacology and optogenetics

To induce pharmacological block of thalamic activity, a glass pipette was filled with a solution containing fluorescent muscimol (Muscimol, BODIPY TMR-X Conjugate, Thermo Fisher) 0.5 mM dissolved in 1% DMSO and ACSF. A small positive pressure was applied to infuse between 250-500 nL into the thalamus. Using the same pipette, we collected the MUA and the effect of muscimol silencing was controlled for by looking at the almost complete loss of MUA spikes. The muscimol diffused for about 20 - 40 minutes and the effect on whisker stimulation was further confirmed by the dramatic decreased of MUA spikes in the thalamic activity following whisker deflection. The animal was then sacrificed and correct positioning of the glass pipette for pharmacological application was confirmed by imaging the fluorescent conjugate of muscimol in fixed tissue using the upright fluorescent microscope Olympus BX51 coupled with Neurolucida software.

A continuous wave solid-state laser source (Cobolt, Vretenvägen, Sweden) was used to deliver yellow (λ = 594 nm, stimulus duration of 500 ms) light illumination through an optical fiber (diameter 200 μm, NA: 0.22, AMS Technologies, Milan, Italy). Laser power was measured at the fiber tip and set at $\sim$30 mW.

QUANTIFICATION AND STATISTICAL ANALYSIS

Data selection

To ensure that our analysis was restricted to synchronized periods of activity, in consecutive 5 s trials we computed the synchronization index, i.e., the ratio between the LFP signal power in low-frequency ([0.1, 4] Hz) over high-frequency bandwidth ([4, 100] Hz) [23, 78]. We ran our analysis only on those trials showing synchronization index > 4 [78].

Up- and down-state detection from LFP signal

Up- and down-states were detected from the cortical LFP signal using the same method described in [22]. Briefly, the raw LFP signal was first low-pass filtered below 500 Hz (using an elliptic filter) and then down-sampled to 1 kHz. Up- and down-states were then detected from the filtered LFP using a method based on combining the approach of Saleem and colleagues [7] (based on the instantaneous phase of the LFP in the low-frequency < 4 Hz band), with a modified version of the algorithm proposed by Mukovski [23] (exploiting differences in beta and gamma-band power between up- and down-states). As described in [22] we optimized the parameters of the algorithm for our experimental conditions by using six simultaneous LFP and patch-clamp recordings on pyramidal neurons. The Saleem method depends on the choice of a few low-frequency bands, whose instantaneous phase is used for state detection, as well as on the corresponding angular parameters that may vary according to the specific recording configuration. We found that under our conditions the optimal LFP bands for state detection were [0 – 1 Hz] and [1 – 2 Hz]. This choice allowed us to maximize the performance of the detection algorithm (data not shown). The output of the Saleem method is a decision (or evidence) variable $S_{delta}(t)$, computed by combining the differential likelihood of observing an up- or down-state from the chosen bands, which varies between 0 and 1 and can be used to determine the instantaneous state. To also take advantage of the information about the state given by higher frequencies, following [23] we combined $S_{delta}(t)$ with another decision variable extracted from the LFP in the 10 – 50 Hz range including the beta and low gamma bands ($S_{beta-gamma}(t)$). To calculate $S_{beta-gamma}(t)$, we first processed the filtered signal to calculate the standard deviation (i.e., root mean square) of the filtered signal in the [10, 51] Hz band in a running frame of 5 ms. We then smoothed the obtained trace with a 50 ms running frame linear filter [23]. Finally, the resulting signal was normalized between 0 and 1, excluding the top 5th percentile, and averaged with $S_{delta}(t)$ to obtain $S_{comb}(t)$. Since the performances of the algorithm considering $S_{comb}(t)$ were slightly higher than when using $S_{delta}(t)$ alone [22], we decided to use $S_{comb}(t)$ instead of $S_{delta}(t)$ to estimate the state at each time instant. To determine the thresholds for the detection of up/down-states, the distribution of $S_{comb}(t)$ (excluding the top 5th percentile) was fitted by a mixture of three Gaussians using an expectation maximization algorithm [7, 22]. Each Gaussian represents a different cortical state: up (highest values of S_{comb}), down (lowest), and indeterminate (intermediate). Time samples corresponding to $S_{comb}(t) > \mu_{UP} - 2\sigma_{UP}$ were assigned to up-states, and samples corresponding to $S_{comb}(t) < \mu_{DOWN} + 2\sigma_{DOWN}$ to down-states (where means and variances of the Gaussians were represented as μ_{UP}, μ_{DOWN}, and σ_{UP}, σ_{DOWN} for the up- and down-states, respectively). The remaining samples were considered as indeterminate state. We set the minimum state duration equal to 100 ms and the minimum inter-state interval equal to 50 ms [22].

After muscimol injection in the thalamus, we re-fitted $S_{comb}(t)$ distributions and determined new up/down-state detection thresholds. We ensured that thresholds in the control condition were not significantly different from thresholds in the muscimol condition for the same animals (data not shown). All the analyses were performed by using custom-made software implemented in MATLAB (The Mathworks, Natick, MA, USA).

Spike detection

We detected action potentials in cortical PV interneurons from juxtasomal recordings by applying a hard-threshold algorithm to the high-pass filtered signal (elliptic filter, cut-off frequency 300 Hz). The threshold was computed as 6 times the estimated standard deviation of the signal. The time stamps of the detected spikes were assigned to the action potential positive peak. Extracellular spikes in the thalamic MUA were detected using a previously published spike detection algorithm known as precise timing spike detection (PTSD) [73]. Briefly, after band-pass filtering (elliptic filter, bandwidth [300, 3000 Hz]), the algorithm looked for pairs of relative maxima/minima in the filtered signal within a user-defined peak lifetime period (set at 3 ms) exceeding a differential threshold set at 8-9 times the estimated standard deviation of the noise. A refractory period of 1 ms was also applied to discard overlapping spikes.

The time stamps of the detected spikes were assigned to the negative voltage peaks. The VPM nucleus contains one type of excitatory neuron the thalamo-cortical cell [79] and virtually no local interneurons [80]. The MUA signal should thus provide a proxy of the integrated activity of VPM thalamo-cortical cells. However, previous work demonstrated that extracellular electrodes placed in the VPM can record narrow spikes generated by the axons of TRN inhibitory cells [81]. Thus, we cannot exclude that part of the VPM MUA signal recorded under our experimental conditions reflects TRN activity.

Phase locking analysis between cortical PV interneurons / thalamic activity and LFP

To investigate the temporal relationship between PV interneurons' spiking activity/thalamic MUA and up/down-state occurrence during spontaneous activity, we asked whether the LFP slow oscillation phase (which in turn reflected cortical state) was related to the occurrence of spikes by quantifying phase locking of recorded spikes. To do that, we applied a previously described method [22, 82, 83]. We computed the instantaneous low-frequency phase of the LFP as the angle of the Hilbert transform of the LFP trace filtered in the [0.1, 4] Hz band. The phase of firing distribution quantifies, for each cell, the phase values at which each spike was fired. Non-uniform phase of firing distributions meant that neurons fired preferentially at certain phases. Hence, phase locking can be detected by assessing departure from uniformity in the distribution of rescaled phases observed at spike times. To correct for the effect of possible non-uniformities in the phase distributions due to asymmetries in the LFP wave shape [83], we made the overall distribution of phase across all time points uniform by rescaling it by its cumulative distribution. The significance of phase locking was computed as departure from uniformity of the phase of firing distribution, using the Rayleigh's test [83]. As a measure of strength of locking, we considered one minus the circular variance, to quantify the concentration of the distribution of angles [82, 84]. We also determined the preferred phase of firing by calculating the circular mean of the phase of firing distribution. To quantify how spikes are distributed during the course of either up- or down-states, we also computed the phase of firing distribution of PV interneuron spikes separately for either up- or down-states. PV spikes recorded during up- (or down-) states were limited to the 5th-95th percentile up-state (or down-state) phase range, i.e., [95, 255] degrees for up-, and [295, 75] degrees for down-state, respectively. In this case, to account for the non-uniformity of the overall distribution of up-state (or down-state) phase, we rescaled it by its cumulative distribution in the above mentioned range. The phase of firing distribution during up- or down-states can be easily compared to the time of firing distribution when normalized to state extremes (0 = start of up/down-state; 1 = end of up/down-state). Point-wise comparison between the phase and time distributions demonstrated no significant difference between the phase and time domain with the exception of the last three bins at the end of the down-state for VPM (marked by asterisks in Figure S1F, paired Student's t test, FDR correction for multiple comparisons). When comparing relative time of firing distributions with the uniform distribution, we observed significant differences only in down-state for both PV interneurons and VPM, with the exception of one point at the end of the up-state for PV cell activity (marked by hashtags in Figures S1E and S1F, left- or right-tailed one-sample Student's t test, FDR corrected for multiple comparisons). Cortical PV interneurons tended to spike more than expected from a uniform distribution at the beginning and less in the middle of the down-state, showing a median time-of-firing of 0.39 ± 0.03 (significantly different from 0.5, one-sample Student's t test, p = 7.71E-4, n = 18 cells in 7 animals). The thalamus showed lower spiking probability in the middle of the down-state and symmetrically higher probability at the borders. Nonetheless, the VPM median time-of-firing was 0.48 ± 0.02 (n = 18 recordings in 7 animals), not significantly different from 0.5 (one-sample Student's t test, p = 0.30, n = 18 in 7 animals). During the up-state, both PV interneurons and the thalamic VPM nucleus fired uniformly, showing median time of firing of 0.49 ± 0.01 and 0.51 ± 0.01, respectively (not significantly different from 0.5, one-sample Student's t test, p = 0.43 and p = 0.45, n = 18 in 7 animals).

Spike-triggered thalamic instantaneous firing rate

To measure the temporal relationship between cortical PV interneuron spiking activity and thalamic MUA during cortical down-states, we computed the spike-triggered VPM instantaneous firing rate (IFR) based on the timing of PV spikes recorded during down-states (this analysis was also extended to PV spike times during up-states, see Results). To estimate the IFR function, we convolved VPM spike trains with Gaussian kernels of increasing standard deviations (1.25 ms – 12.5 ms). We averaged VPM IFR functions in time windows of corresponding increasing durations (10-100 ms) preceding (pre-spike) and following (post-spike) PV spikes in down-state. We finally selected those parameter values that maximized the correlation between VPM and PV activity in the pre-spike window (Gaussian kernel standard deviation of 12.5 ms, corresponding to low-pass filtering with 20-Hz cut-off frequency, and pre- / post-spike time windows of 100 ms). For each PV interneuron, to test whether the VPM mean firing rate preceding PV spikes in down-state was higher than expected by chance, we repeated the same analysis for "surrogate" PV spikes. We generated 100 surrogate datasets by randomly sampling PV-silent down-states while preserving the total number of spikes and the phase of firing distribution in down-state. We checked that, by doing this, also the time of firing distribution during down-state, when normalized to state extremes, was approximately preserved. We also fixed the maximum number of sampled down-states and the maximum number of spikes *per* down-state. This helped us to also mimic with our surrogate data the statistical distribution of the number of spikes *per* down-state. We then computed for each surrogate dataset the VPM IFR triggered by surrogate spikes and computed preceding and following VPM mean firing rates, as done for the actual PV spikes. For each PV interneuron, we computed the z-score of the average VPM firing rate in the pre-spike and post-spike windows with respect to the corresponding statistical distribution of surrogate mean firing rates to check for significant statistical differences between actual and surrogate data. Moreover, we asked whether the statistical distribution of VPM mean firing rate in the pre-spike window was significantly higher than in the post-spike

window, suggesting a possible temporal relationship directed from VPM to PV interneurons in down-state. The latter analysis was also performed for VPM mean firing rate preceding and following PV spikes in up-state.

Evoked down-to-up transition latency

We computed the latencies of down-to-up transitions evoked by optogenetic suppression of PV interneurons during cortical down-states both under control conditions and after muscimol injection in the thalamus in two steps. First, for finding putative down-state periods, we slightly modified the previously described detection algorithm by simplifying it, since we did not need to determine precisely up/down-state boundaries in this case. We observed a short polarization of the signal at the beginning of the optogenetic stimulation, which can be considered as an artifact of the illumination (duration $\sim$ 4 ms). To correct for this we substracted to the LFP signal the mean of all stimulation trials in which the artifact was visible and not superimposed to a fast evoked down-to-up state transition, and finally smoothed it using a 14-ms window moving average filter in a 60-ms window centered on the stimulation onset. We then computed the evidence (or decision) variable $S_{beta-gamma}(t)$ as previously described (using 10-ms windows for running standard deviation computation of the [10, 51] Hz filtered signal,) and we detected as putative down-states those time periods in which the processed signal was lower than its overall median (see Figure S4A, middle trace). The same thresholds computed under control conditions were used to detect putative down-states after thalamic pharmacological inhibition. We then selected those 5 s trials in which the onset of the optogenetic stimulation fell in a putative down-state and we computed the discrete temporal derivative of the LFP signal (Figure S4A, bottom trace). For each optogenetic stimulation falling in a down-state, we computed the mean μ and standard deviation σ of the LFP derivative in the corresponding down-state. We identified the evoked transition to an up-state as the time sample when the LFP derivative exceeded the threshold T defined as $T = \mu - k\sigma$, with k being an arbitrary multiplicative constant set at 4. The latency of the transition was therefore computed as the time interval between the onset of the illumination period and the first crossing of the threshold T (Figure S4A, top trace). To check whether light-evoked transition latencies were shorter than expected by chance due to optogenetic inhibition of PV cells, we selected periodically distributed points during spontaneous activity as "surrogate" stimulation onsets and performed the same analysis by computing the latencies to the next spontaneous down-to-up transitions (setting $k = 2$ to account for slower spontaneous transition slopes with respect to light-evoked ones). We considered as *spontaneous* the LFP activity occurring at least 50 ms before the onset and 550 ms after the offset of each illumination period in each 5 s trial. To control for possible long-term effects of the optogenetic PV inhibition in cortical activity, we compared the latencies of spontaneous down-to-up transitions obtained with this method with latencies calculated on longer recordings of fully spontaneous activity for 20 different random selection of the beginning of stimulation time instants in 3 animals under control conditions and after muscimol injection. The obtained results were not significantly different from previous ones (data not shown). Then, we compared the obtained results by using different thresholds for putative down-state identification from $S_{beta-gamma}(t)$ distribution, instead of using the signal's median. (i.e., the full-width-half-maximum of the first peak of $S_{beta-gamma}(t)$probability distribution function, and the mean plus standard deviation of the first Gaussian component of two-Gaussian mixture fitting of $S_{beta-gamma}(t)$ distribution). In both cases, we obtained statistically indistinguishable results compared to the median threshold (Figure S4B).

Finally, we developed a linear mixed effects model (LME) [85] to further explore the effects that pharmacological inhibition of the thalamus had on the down-to-up state transition regulatory function of PV cells. This statistical model allowed us to discriminate the contributions to the variance of the experimental variables of interest (fixed effects) from the ones due to animal variability (random effects). We defined as "fixed effects" pharmacological *treatment* (considering either control or muscimol conditions), and *stimulation* (considering either optogenetically induced transitions or spontaneous ones), and we also considered their interaction. To account for animal variability, we first included as "random effects" the *animal identity*, and variations in intercept among animals within both *treatment* and *stimulation*.

The model is described by the following equation:

$$Latency_{ijk} = \beta_0 + \beta_1(i) + \beta_2(j) + \beta_3(i,j) + \gamma_{0k} + \gamma_{1k}(i) + \gamma_{2k}(j) + \varepsilon_{ijk}$$

with β the fixed effects, γ the random effects, for *treatment* i, *stimulation* j and *animal* k. We first tested the statistical significance of each random effect by using a likelihood ratio test (LRT). The LRT compared the likelihood of the complete model with reduced ones dropping one effect at a time. The random intercepts corresponding to the interaction between animal and treatment and between animal and stimulation were significant (γ_{1k}; p = 0.02, γ_{2k}; p = 1.4E-8), but animal identity was not and we therefore excluded it from the model (γ_{0k}; p = 0.26). Then, after re-fitting the model, each fixed effect was evaluated using type II Wald chi-square test [86]. To evaluate the output of this model, we performed a pairwise contrast test between the means of the factorial groups adjusting the degrees of freedom using the Kenward-Roger method and the p values using the Tukey method for comparing a family of four estimates (Figure S4C).

Statistics

The Kolmogorov-Smirnov test was run on each experimental sample with N > 5 to test for normality. Two-tailed (unless otherwise stated) Student's t test (in case of normal distribution), and the Wilcoxon rank-sum or signed-rank (for unpaired and paired comparison of non-normal distributed data, respectively) tests were used when comparing two populations. For comparison of more than two populations, one-way ANOVA was used. We used Benjamini-Hochberg procedure to correct p values for FDR in case of multiple comparisons, and binomial test to combine p values resulting from several independent tests bearing upon the same overall

hypothesis. To study the significance of fixed effects in the LME model, we used the Type II Wald Chi-square test and a pairwise contrasts test adjusted with the Tukey method to compare the population means of the model. When not specified explicitly, the significance level for statistical testing was set at 0.05. Statistical analysis was performed using Origin 2016, RStudio, or MATLAB software.

DATA AND SOFTWARE AVAILABILITY

The raw datasets (i.e., electrophysiological recordings) and custom MATLAB code were deposited in Zenodo. The DOI to the deposited data and code reported in this paper is https://doi.org/10.5281/zenodo.2609157.

Current Biology, Volume *29*

Supplemental Information

Thalamic Drive of Cortical Parvalbumin-Positive Interneurons during Down States in Anesthetized Mice

Stefano Zucca, Valentina Pasquale, Pedro Lagomarsino de Leon Roig, Stefano Panzeri, and Tommaso Fellin

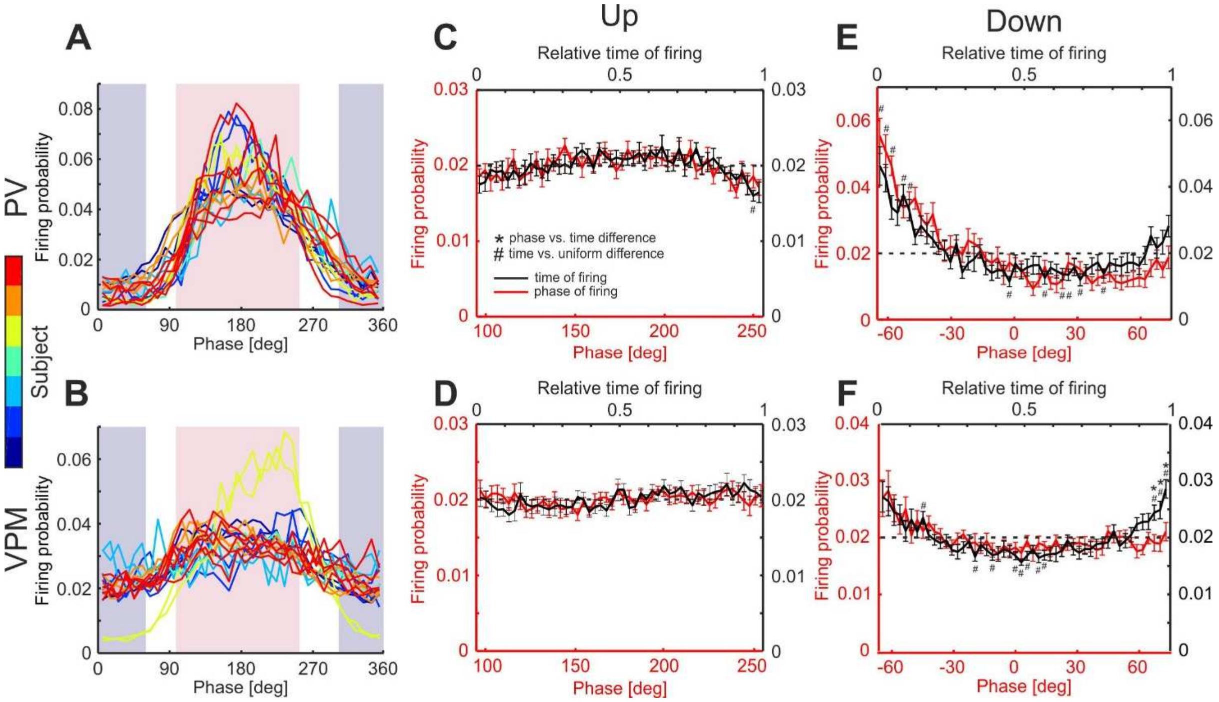

Figure S1. Phase-of-firing and relative time-of-firing distributions of PV cells and VPM MUA during cortical up- and down-states. Related to Figure 1. (A-B) Phase-of-firing distributions of all PV interneurons (color-coded by animal (A) and simultaneously recorded VPM MUA (B). **(C-D)** Phase-of-firing distribution (red, mean ± s.e.m., n = 18 cells in 7 animals) and relative time-of-firing distribution (black) for PV interneurons (C) and VPM MUA (D) during cortical up-states. **(E-F**) Same as for (C, D), but for cortical down-states (mean ± s.e.m., n = 18 recordings in 7 animals). For all panels: asterisks indicate point-wise significant statistical differences between the two curves (paired Student's t-test, FDR corrected for multiple comparisons, p-level = 0.05). Hashtags indicate point-wise significant statistical differences between time-of-firing distributions and the uniform distribution (one-sample Student's t-test, FDR corrected for multiple comparisons, p-level = 0.05).

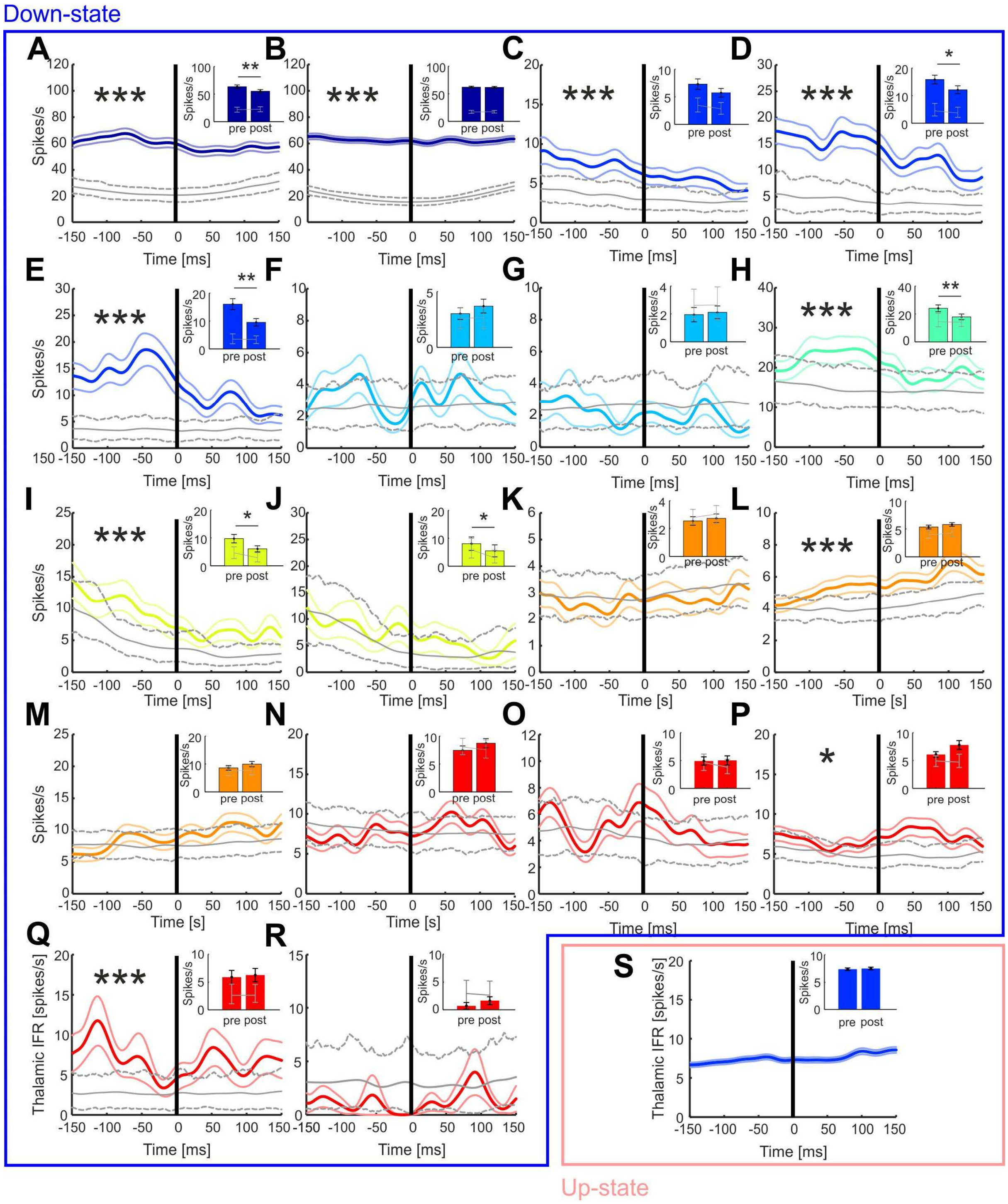

Figure S2. Spike-triggered average analysis across individual experiments. Related to Figure 2. (**A-R**) Spike-triggered VPM firing rate based on PV interneuron spike times during cortical down-states for all recorded cells (n = 18 from 7 animals, Gaussian kernel standard deviation 12.5 ms, mean ± s.e.m.). Statistical difference of the pre-spike VPM mean firing rate with respect to surrogate data is indicated by the asterisks (z-test, $p < 0.05$). Insets: VPM firing rate before and after a PV spike in down-state (pre: [-100, 0] ms; post: [0, 100 ms]; mean ± s.e.m.; Wilcoxon signed rank test). **(S)** Same as in (A-R**)** but for PV interneuron spike times during cortical up-states for one representative recorded cell.

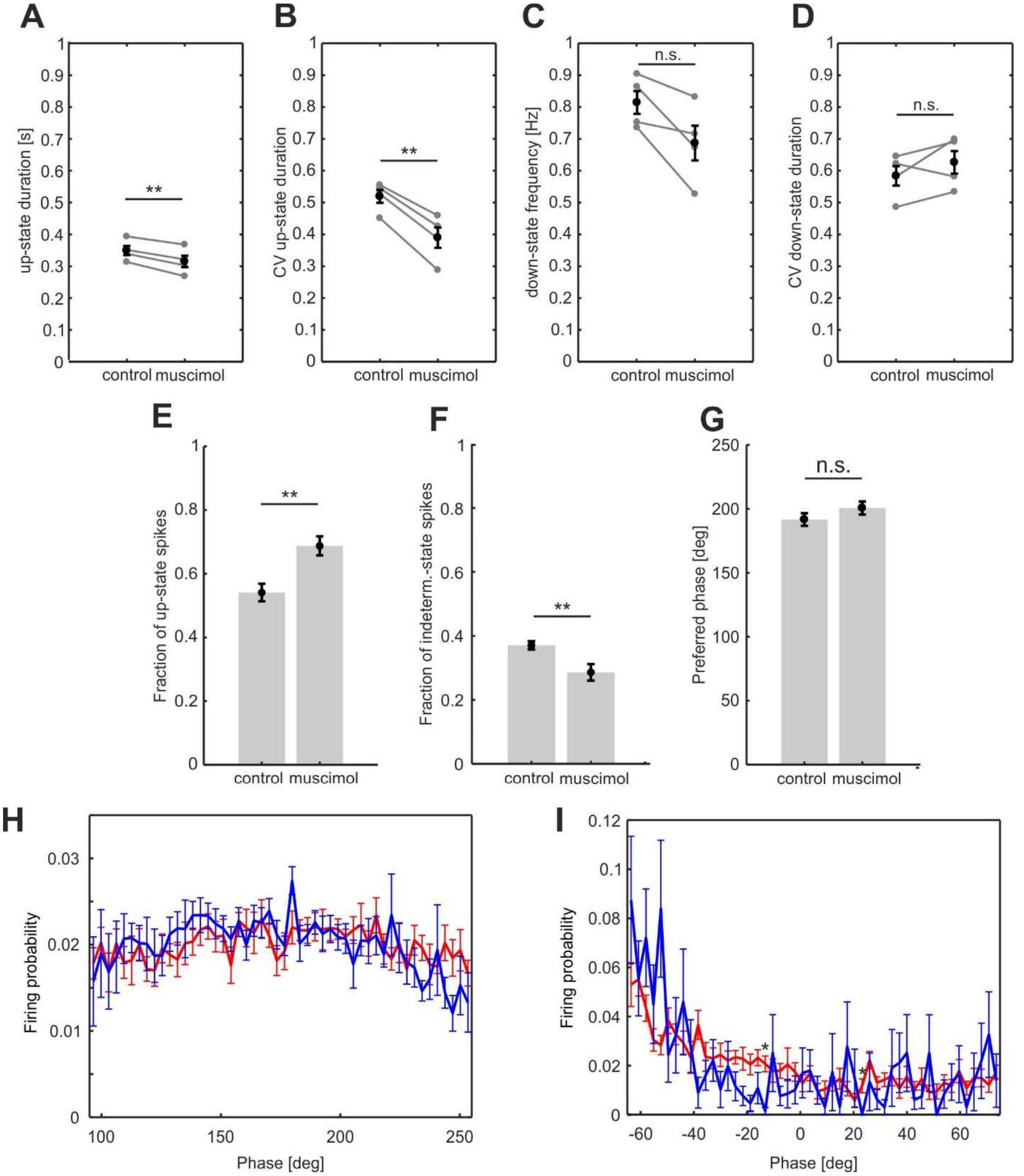

Figure S3. Effect of thalamic muscimol injection on cortical up and down-state dynamics and PV interneuron firing activity. **Related to Figure 3. (A-B**) Up-state duration (A) and its coefficient of variation (B) under control conditions and after muscimol injection in the thalamus (mean ± s.e.m., paired Student's t-test, p = 0.009 for A and p = 0.003 for B, n = 4 animals). **(C**) Down-state frequency under control conditions and after muscimol injection in the thalamus (mean ± s.e.m., p = 0.056, paired Student's t-test, n = 4 animals). **(D**) Coefficient of variation of down-state duration under control conditions and after muscimol injection in the thalamus (mean ± s.e.m., paired Student's t-test p = 0.27 for D, n = 4 animals). **(E-F**) Fraction of up (E) and indeterminate (F) state spikes under control conditions and after muscimol injection (mean ± s.e.m., Student's t-test, p = 0.003 for C and p = 0.008 for D, control: n = 8 cells, muscimol: n = 7 cells from 4 animals). **(G)** Mean phase of firing of PV interneurons under control conditions and after thalamic muscimol injection (circular mean ± s.e.m, p = 0.26, Watson-Williams test, control: n = 8, muscimol: n = 7 from 4 animals). **(H-I**) Phase-of-firing distribution of PV interneurons during cortical up (H) and down (I) states under control conditions (red, mean ± s.e.m., n = 8 cells in 4 animals) and after thalamic muscimol injection (blue, mean ± s.e.m., n = 7 cells in 4 animals). In both panels, the two curves do not show significant point-wise differences, except for two points (marked by asterisks, Student's t-test, FDR corrected for multiple comparisons, p-level = 0.05).

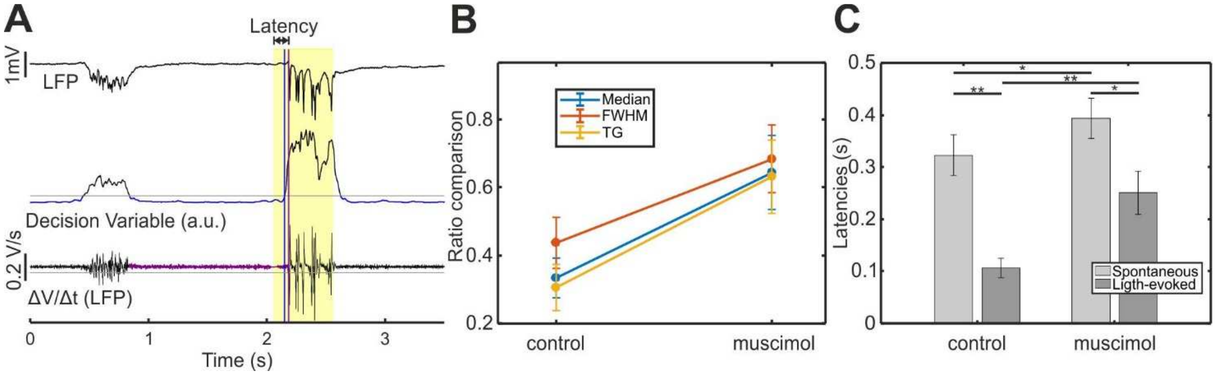

Figure S4. Latencies of down-to-up transitions upon inhibition of cortical PV interneurons under control condition and after thalamic pharmacological inactivation. Related to Figure 4. (**A**) Representative cortical LFP trace after muscimol injection in the thalamus (top) showing characteristic silent and active periods during slow oscillation network dynamics. The gamma-filtered signal (middle) is used to detect down-states (blue periods). This detection is then used to select the traces in which the beginning of the optogenetic inhibition of PV cells (yellow bar) occurred in a down-state. The transition to an up-state (purple vertical line) is defined as occurring when the LFP temporal derivative (bottom) crosses the threshold T (gray line). The latency of the transition is therefore the time interval between the beginning of the yellow bar and the purple vertical line. (**B**) Comparison of the average latency ratios calculated using three different thresholds (median, full-width-half-maximum and two-Gaussian mixture fitting, indicated by different colors) for down-state detection (see STAR Methods) (mean ± s.e.m., one-way ANOVA, p = 0.36 for control and p = 0.94 for muscimol, n = 7 animals). **(C**) Latencies of spontaneous and light-evoked down-to-up transitions under control conditions and after muscimol application in the thalamus (mean ± s.e.m., n = 7 animals).
